# Conservation Mitonuclear Replacement: Facilitated mitochondrial adaptation for a changing world

**DOI:** 10.1111/eva.13642

**Published:** 2024-03-10

**Authors:** Erik N. K. Iverson

**Affiliations:** ^1^ Department of Integrative Biology The University of Texas at Austin Austin Texas USA

**Keywords:** assisted evolution, climate change, conservation, facilitated adaptation, genetic rescue, mitochondria, mitonuclear interactions

## Abstract

Most species will not be able to migrate fast enough to cope with climate change, nor evolve quickly enough with current levels of genetic variation. Exacerbating the problem are anthropogenic influences on adaptive potential, including the prevention of gene flow through habitat fragmentation and the erosion of genetic diversity in small, bottlenecked populations. Facilitated adaptation, or assisted evolution, offers a way to augment adaptive genetic variation via artificial selection, induced hybridization, or genetic engineering. One key source of genetic variation, particularly for climatic adaptation, are the core metabolic genes encoded by the mitochondrial genome. These genes influence environmental tolerance to heat, drought, and hypoxia, but must interact intimately and co‐evolve with a suite of important nuclear genes. These coadapted mitonuclear genes form some of the important reproductive barriers between species. Mitochondrial genomes can and do introgress between species in an adaptive manner, and they may co‐introgress with nuclear genes important for maintaining mitonuclear compatibility. Managers should consider the relevance of mitonuclear genetic variability in conservation decision‐making, including as a tool for facilitating adaptation. I propose a novel technique dubbed Conservation Mitonuclear Replacement (CmNR), which entails replacing the core metabolic machinery of a threatened species—the mitochondrial genome and key nuclear loci—with those from a closely related species or a divergent population, which may be better‐adapted to climatic changes or carry a lower genetic load. The most feasible route to CmNR is to combine CRISPR‐based nuclear genetic editing with mitochondrial replacement and assisted reproductive technologies. This method preserves much of an organism's phenotype and could allow populations to persist in the wild when no other suitable conservation options exist. The technique could be particularly important on mountaintops, where rising temperatures threaten an alarming number of species with almost certain extinction in the next century.

## 1 INTRODUCTION

Conservationists increasingly recognize the utility in identifying adaptive or potentially adaptive genetic variation for long‐term genetic management of populations in a changing climate (Harrisson et al., 2014; Hoelzel et al., 2019; Mable, 2019; Schaffer et al., 2022). Adaptive genetic variation, or the *additive genetic variance in fitness* (sensu Fisher, 1930), is the total amount of allelic diversity that might be selected upon by environmental variables such as temperature, precipitation, pollution, ocean acidification, and disease, among others. Genetic diversity is extremely important for population health and is correlated with adaptive potential due to the polygenic nature of most traits (Kardos et al., 2021; Ørsted et al., 2019); however, populations with equivalent neutral diversity may still differ in the presence and frequency of adaptive or potentially adaptive variants relevant to different conservation challenges (Mable, 2019). Studies have begun to map and identify genome‐wide variants correlated with the present distribution of environmental variation and create predictive genomic models of vulnerability (e.g., Bay et al., 2018; Chen, Grossfurther, et al., 2022; Chen, Jiang, et al., 2022; Hanson et al., 2020; Ruegg et al., 2018), although the functional linkage between genotype and phenotype remains difficult. Determining the identity, function, and interactions of adaptive alleles is a major opportunity for the expansion of genetic rescue efforts and conservation interventions (Funk et al., 2012, 2019; Whiteley et al., 2015).

Intrinsic or anthropogenic limits on dispersal make climate‐tracking by many species difficult or impossible (Feeley et al., 2011; Forero‐Medina et al., 2011; Freeman et al., 2018; Nowak et al., 2022), and present levels of standing genetic diversity are likely inadequate for either thermal tolerance (Bennett et al., 2021) or phenology (Radchuk et al., 2019; Vtipil & Sheth, 2020) to evolve under natural selection fast enough for most species to adapt to climate change. The challenge of adaptation is exacerbated in species with small effective population sizes, where adaptive potential may be low and deleterious alleles can be exposed via inbreeding (Aitken & Whitlock, 2013; Hoffmann & Sgró, 2011). In addition, habitat fragmentation and barriers to dispersal act to reduce gene flow among populations, preventing the exchange of adaptive alleles. Faced with these threats, adaptive introgression via hybridization with other populations—and species—may be the only evolutionary mechanism that can allow many populations to adapt to anthropogenic stressors on meaningful timescales (Baskett & Gomulkiewicz, 2011; Chunco, 2014; Hamilton & Miller, 2016; Stelkens et al., 2014; Vedder et al., 2022).

Facilitated adaptation, or assisted evolution, comprises human‐directed genetic aid to wild populations that manipulates the frequencies of adaptive alleles for a conservation purpose. This might take the form either of selective introgression of known adaptive variants, manipulating fitness outcomes in wild populations (as through head‐starting, culling, or otherwise artificially‐selecting particular genomes), or genetic editing (Chakravarti & van Oppen, 2018; Kosch et al., 2022; Phelps et al., 2020; Samuel et al., 2020; Thomas et al., 2013; Van Oppen et al., 2015). In one case, facilitated adaptation has already endowed a species with a novel ability to persist in the face of anthropogenic change: both interspecific hybridization and genetic engineering have been used to give American chestnut trees (*Castanea dentata*) genes necessary to resist a devastating introduced blight, and the species is slated for imminent reforestation across its former range (Newhouse & Powell, 2021; Steiner et al., 2017; Worthen et al., 2010). Disease resistance is highly polygenic and variably expressed in hybrids, but is effectively conferred by a single locus in transgenic trees. In general, one of the main hurdles to facilitated adaptation is identifying the number, identity, and architecture of relevant adaptive loci (Cleves et al., 2020; Thomas et al., 2013; Westbrook et al., 2020). If adaptive loci can be identified and their functions understood, more‐targeted genetic interventions are possible that are less disruptive to native genetic variation and locally adapted phenotypes. Even where traits are controlled by many loci, manipulation of one or several genes can significantly shift fitness. For instance, heat tolerance is highly polygenic in cattle (Freitas et al., 2021), but editing of one locus can confer meaningful performance improvements (“CRISPR beef cattle get FDA green light,” 2022; Eisemann et al., 2020; Landaeta‐Hernández et al., 2021).

The mitochondrial (mt) genome encodes genes for the core energetic machinery of eukaryotes and influences metabolic rate, the cellular stress response, and the immune response, among other highly integrative phenotypes (Aguilar‐López et al., 2020; Arnqvist et al., 2010; Brown et al., 2020; Hill, 2014). The mt genome was originally treated as a neutral genetic marker most useful for barcoding and phylogenetics; however, this paradigm has long been seen as unsatisfactory (Ballard & Kreitman, 1995; Ballard & Rand, 2005; Hurst & Jiggins, 2005), and the mt genome is increasingly recognized as a subject of adaptive evolution (Hill, 2019a; James et al., 2016). Experiments demonstrate that the mt genome accounts for a disproportionately large share of heritability in integrative eco‐physiological traits such as heat‐, desiccation‐, hypoxia‐, and starvation‐resistance (Camus et al., 2017; Chung & Schulte, 2020; Immonen et al., 2020; Lajbner et al., 2018; Lasne et al., 2019).

Despite recognition of its important role in adaptation, several characteristics of the animal mt genome—including uniparental inheritance, a lack of recombination, and strong purifying selection—also act to reduce standing genetic variation and adaptive potential in mt genes (Comeron et al., 2008; Neiman & Taylor, 2009; Rand, 2008; Stewart et al., 2008). Mt genomes are also subject to low effective population sizes, experience strong drift, and have a tendency to fix deleterious substitutions (Lynch & Blanchard, 1998; Nachman, 1998), a process that can lead to substantial genetic load in small populations. Adaptation in mt genes is further constrained by their need for tight co‐adaptation with nuclear genes (Rand et al., 2004); genes from each genome are adapted both to the environment and to each other, causing complex Gene × Gene × Environment interactions (Arnqvist et al., 2010; Dowling et al., 2007; Rand et al., 2018). Thus, while many estimates already suggest that evolution cannot keep pace with climate change, the problem is compounded for adaptive evolution in the mt geneome, where drift, linkage, and mitonuclear compatibility pose challenges to the effectiveness of selection.

In natural populations, mt genotypes that are less fit – either because of poor fit to the environment or because of the accumulation of deleterious alleles – can be replaced by more fit haplotypes from neighboring populations or even from other species (Sloan et al., 2017; Toews & Brelsford, 2012). This naturally occurring adaptive introgression suggests that novel mt haplotypes might be intentionally introgressed by conservationists for their beneficial effects on population fitness. However, coevolution between mt and nuclear genes within populations can lead to damaging mitonuclear incompatibilities in hybrids, which are a major source of reproductive isolation between species (Burton & Barreto, 2012; Du et al., 2017; McFarlane et al., 2016; Moran et al., 2022). Where populations have adapted to divergent climates, the degree of mitonuclear incompatibility might be heightened (Frankham et al., 2011; McFarlane et al., 2016; Moran et al., 2022; van der Heijden et al., 2019; Yamaguchi & Otto, 2020). Thus, if mt genetic variation is to be introduced intentionally for its adaptive benefits, the challenge is to anticipate, prevent, or ameliorate mitonuclear incompatibilities.

I present a novel proposal for facilitated adaptation to enable the persistence of threatened species by substituting a set of co‐adapted mt and nuclear alleles from a close relative. This mt‐donor population may already be well‐adapted to present or future climatic conditions threatening a species, or it may simply be uncorrupted by excessive mt mutational load. The promise of this technique lies in the underappreciated adaptive significance of mt genes and the role of key mt‐interacting nuclear loci (N‐mt genes) in allowing successful introgression of better adapted—or less maladapted—haplotypes. I first discuss the evidence for relevant adaptive variation among mt haplotypes and the basis in mitonuclear theory for a successful, modular replacement of critical energetic machinery. I then discuss the advantages of replacing a species' mt genome with biotechnological approaches rather than through natural or facilitated hybridization, and suggest a way forward based on existing technologies. The technique, which I call Conservation Mitonuclear Replacement (CmNR), is detailed further in the supplements. Finally, I discuss the ecological, evolutionary, and ethical consequences and conservation‐relevance of the technique, were it to be applied.

## 2 MITONUCLEAR CO‐ADAPTATION TO CLIMATE

Mitochondrial genes appear to play an outsized role in climatic adaptation relative to their small proportion of the eukaryotic genome (Lasne et al., 2019). Within species, mt haplotypes commonly map onto climatic differences within a species' range (Camus et al., 2017; Mishmar et al., 2003; Rank et al., 2020; Silva et al., 2014; Wang, Ju, et al., 2021; Wang, Ore, et al., 2021; Zorzato et al., 2022), and experiments have demonstrated the importance of mt variation for metabolic phenotypes and thermal adaptation in both ectotherms and endotherms (Harada et al., 2019; Lajbner et al., 2018; Pichaud et al., 2012; Toews et al., 2014). Aspects of mt function with evidence of climate‐imposed selection include the functional efficiency of the individual mt electron transport system (ETS) complexes (Dingley et al., 2014; Harada et al., 2019; Pichaud et al., 2012) including in particular Complex I (Moran et al., 2022) and Complex IV (Chung et al., 2017; Scott et al., 2011), the efficiency of mt replication and transcription and the quantity of mt gene products (Bar‐Yaacov et al., 2015; Camus et al., 2017; Scott et al., 2018), the degree of uncoupling in the ETS (Stier, Bize, et al., 2014; Stier, Massemin, & Criscuolo, 2014), the degree of reactive oxygen species (ROS) production and/or sensitivity to ROS levels (Dingley et al., 2014), the propagation of the cellular stress response (Sokolova, 2018), the shape, fluidity, and permeability of mt membranes (Dingley et al., 2014), the number and volume of mitochondria (Cheng et al., 2013; Johnston et al., 1998; Scott et al., 2018), their rate of turnover (Sokolova, 2018), and their intra‐cellular position (Scott et al., 2018).

Crucially, the fitness of mt variants is influenced by both the environment and the nuclear genetic background (Arnqvist et al., 2010; Dowling et al., 2007; Immonen et al., 2020; Rand et al., 2018; Rank et al., 2020). In all of the domains listed above, and in many other intracellular processes, mismatches between non‐co‐adapted mt and N‐mt genes may reduce fitness in some or all environments (Hill, 2019a; Table 1). For instance, mitonuclear interactions influence metabolic phenotypes like metabolic rate (Arnqvist et al., 2010; McFarlane et al., 2016), growth rate (Nagao et al., 1998; Rand et al., 2021), BMI (Ludwig‐Słomczyńska et al., 2020) and other metabolic traits (Kraja et al., 2019), and different mitonuclear combinations are favored on different diets (Camus et al., 2020; Mossman et al., 2016; Rand et al., 2021) and in different environments (Morales et al., 2018; Rank et al., 2020; Wang, Ju, et al., 2021; Wang, Ore, et al., 2021). Mitonuclear coadaptation might also be important for adaptation to particular environmental axes in ways distinct from nuclear genes. For instance, in two avian species complexes, mt and N‐mt genetic clines appear to be structured by different environmental factors from those influencing the majority of nuclear loci (Morales et al., 2018; Wang, Ju, et al., 2021; Wang, Ore, et al., 2021). In both of these systems, the major axis of nuclear differentiation is a North/South latitudinal axis, while mitochondrial haplotypes – and nuclear linkage groups appearing to contain many mitonuclear genes – are differentiated instead along an inland/coastal axis corresponding to an aridity gradient. Incompatibilities between mt and N‐mt genes are a source of reproductive isolation (Burton & Barreto, 2012; Gershoni et al., 2009; Havird, et al., 2019; Hill, 2019a, 2019b; Hutter, 2002; Sunnucks et al., 2017), and such incompatibilities between congeners from different climates appear to be linked to a breakdown in metabolic efficiency (McFarlane et al., 2016; Moran et al., 2022; van der Heijden et al., 2019). Because co‐adapted mitonuclear genetic variants are so important in environmental adaptation between related species, they represent key loci to target for facilitated adaptation.

**TABLE 1:** Known and hypothesized nuclear‐mitochondrial compatibility loci relevant to fitness.

Site of mt‐nuclear interaction	Nuclear genes involved	Hypothesized mechanism of fitness loss	Documented effect on phenotype	Examples & support
*Direct interactions*				
OXPHOS complexes	~75 CI, CIII, CIV, & CV protein subunits; Cytochrome C	Poor fit between mt & nuclear complex components, altered conformational states, & reduced proton pumping → low ATP yield, increased ROS production	Elevated metabolic rate; delayed/arrested development; diabetes susceptibility	Du et al. (2017); Ellison et al. (2008); Ellison and Burton (2006); Gershoni et al. (2014); Moran et al. (2022); Sackton et al. (2003)
Mt replication & transcription machinery; post‐transcriptional regulation	*POLG*, *POLG2*, *mtRPOL*, *TFAM*, *TFB1*, *TFB2*, *Twinkle*, ligase; *Cerox1*, *AGO2*	Poor fit of replication enzymes to promoters → Low mt DNA copy number; Poor fit of transcription enzymes to promoters → Low mt mRNA transcript number	Insufficient stress response	Ellison and Burton (2006, 2008, 2010); Zaidi and Makova (2019)
Mt translational machinery	80 ribosomal mt proteins (e.g., *RMP*, *S35*), 17 aminoacyl‐tRNA synthetases, initiation factors, elongation factors; *CCM1* & other pentatricopeptide repeats	Poor fit between mt & nuclear ribosomal components, poor fit of nuclear aminoacyl‐tRNA synthetases for mt tRNAs → low OXPHOS protein expression & efficiency, high ROS	Sterility/infertility; compromised growth & fecundity; Various neo‐natal disorders (reviewed in Burton & Barreto, 2012)	Baris et al. (2017); Hoekstra et al. (2013); Jhuang et al. (2017); Lee et al. (2008); Meiklejohn et al. (2013); Pichaud et al. (2019); Zhang et al. (2017)
Antero/retrograde signaling	*NRF1*, *NRF2*, & other antioxidant response elements; various nuclear promoters; *Tar1p* & other mt‐targeted nuclear signals	Mismatch between mt peptides and nuclear promoters (or vice versa), mismatch between mt small/interfering RNAs and nuclear mRNA targets (or vice versa) → mismatch between mt function and cellular/organismal needs		See Mangalhara and Shadel (2018); Pozzi and Dowling (2021)
*Indirect interactions*				
Regulation of metabolism, including TCA/Krebs cycle	*Mt‐Acyl‐CoA thioesterase‐9*; *Adenylate kinase*; *NAD‐dependent malic enzyme*, *Aim22* (lipoylation)	Mismatch in rate of OXPHOS substrate production → lower OXPHOS efficiency		Baris et al. (2017); Chou et al. (2010)
OXPHOS guide proteins & assembly factors	*ACAD9*, *NDUFA13*, etc.	Misfolding of OXPHOS supernumerary proteins → low ATP yield, increased ROS production	Delayed/arrested development	Bar‐Yaacov et al. (2015); Moran et al. (2022)
Mt membrane construction and uncoupling proteins	*UCP1*, *UCP2*, *UCP3*, *avUCP*	Inappropriate membrane permeability → OXPHOS inefficiency		Not yet reported
Mt ROS sensing and regulation	Cytochrome C, antioxidant response elements	Inappropriate ROS sensing and regulation → premature apoptosis		Not yet reported
Mt methylation	*DNMT3B*, *METTL4* & other DNA methyl‐transferases	Inappropriate methylation → poor immediate and transgenerational plasticity		See Breton et al. (2021)

*Note*: Nuclear genes listed are potential targets for genetic editing to increase mitonuclear compatibility. For many types of interactions, no work has directly linked them to a phenotypic effect, but because many mitonuclear incompatibilities manifest through reduced oxidative phosphorylation (OXPHOS) function, similar phenotypes can be expected to those so far documented. Modified from Burton and Barreto (2012).

## 3 MODULARITY OF THE MITONUCLEAR ENERGETIC MACHINERY

Mitochondria are highly integrated into cellular processes beyond energy production, including apoptosis, calcium‐signaling, and redox reactions (Aguilar‐López et al., 2020; Brown et al., 2020; Hajnóczky et al., 2006). In order to successfully transpose a mt genome between species, mt genes and a manageable number of nuclear genes would need to form a modular system that can be replaced without disrupting other important interactions across the nuclear genome. Evidence for modularity comes from the fact that mt genomes introgress between species relatively frequently in nature (Sloan et al., 2017; Toews & Brelsford, 2012), and N‐mt loci frequently co‐introgress with them against the grain of prevailing nuclear ancestry patterns (see below). While mt introgression may have selectively‐neutral demographic causes (such as sex‐biased dispersal; Toews & Brelsford, 2012), its commonness suggests at a minimum that there are frequent contexts in which the movement of a mt genome into a novel nuclear background is not strongly selected against.

If mitonuclear incompatibilities are present during the initial hybridization, they might be overcome in one of two ways. First, the novel mitogenome might be objectively more fit than the original, perhaps because the original suffered from the accumulation of deleterious mutations or because an environmental change has altered the balance of G × G × E interactions (Sloan et al., 2017). In this case, the fitness benefits of a new mitogenome will outweigh the fitness costs of any mitonuclear incompatibilities (Hill, 2019b). A recent model proposed that mitonuclear discordance, a signal of mt introgression (Toews & Brelsford, 2012), is best explained by direct selection on the mitogenome (Bonnet et al., 2017). Second, the new mitogenome might introgress along with key co‐adapted nuclear genes, such that mitonuclear compatibility is maintained (Sloan et al., 2017). Mitonuclear co‐introgression provides strong evidence that mitonuclear machinery can be substituted in a modular fashion without disrupting other critical genomic interactions. The process has been documented in *Drosophila* (Beck et al., 2015; Box 1), swordtail fishes (Moran et al., 2022), several birds (Hermansen et al., 2014; Morales et al., 2018; Nikelski et al., 2021; Trier et al., 2014; Wang, Ore, et al., 2021), and hares (Seixas et al., 2018), and is debated in macaques (Bailey & Stevison, 2021; Evans et al., 2021; Zhu & Evans, 2023). The implication is that when a mt haplotype moves between closely related species, sufficient mitonuclear co‐adaptation can be maintained if just a few key nuclear genes co‐introgress (e.g., Moran et al., 2022; Seixas et al., 2018), rather than the thousands of nuclear genes with potential interactions. This gives some insight into the number and nature of mitonuclear incompatibilities that cause intrinsic post‐zygotic isolation between closely related species, which might often be few with significant effects rather than many with weak effects. For example, in the case of mitonuclear incompatibility between two swordtail fishes (*Xiphophorus*) living at different water temperatures, robust sequencing has revealed about 5 nuclear loci with mt‐interactions that cause a severe impact on fitness, with two of them co‐introgressing with the mt haplotype (Moran et al., 2022).

Mt introgressions often appear linked to climatic adaptation (Boratyński et al., 2015; Morales et al., 2018; Wang, Ju, et al., 2021; Wang, Ore, et al., 2021). In a well‐documented example, the mt genome of a wide‐ranging temperate cichlid is introgressing into an isolated cichlid species confined to warm springs (Hulsey et al., 2016). There is evidence for relaxed selection on the mitogenome of the isolated species, indicating that its smaller effective population size allowed the accumulation of deleterious mutations. However, the degree of introgression is also related to the temperature of the spring: within colder springs, introgression from the more temperate (cold‐adapted) species is more substantial. This suggests that multiple selective pressures might determine the degree of mt introgression, with favorable environmental conditions facilitating the introgression of mitogenomes that may or may not also be intrinsically more fit. In another example, colonization of colder, more northerly environments by the Eurasian bank vole (*Myodes glareolus*) appears to be linked to mt introgression from a neighboring species (*M. rutilis*) that inhabits the Arctic (Boratyński et al., 2011, 2014). Although adaptive explanations are commonly invoked for mt introgression (Toews & Brelsford, 2012), such claims should be tested against a neutral model of diffusion of the mt genome with distance (as in Hulsey et al., 2016), which could generate a spurious pattern of increasing introgression frequency with climatic similarity to the donor species (Seixas et al., 2018). The co‐introgression of very limited ancestry blocks containing N‐mt genes is strong evidence for adaptive significance to mt introgression and the importance of co‐adaptation with nuclear genes.

## 4 CONSERVATION IMPLICATIONS OF MITONUCLEAR CO‐ADAPTATION AND MODULARITY

### 4.1 Traditional genetic rescue targeting the mitochondrial genome

The recognition that mt & N‐mt genes are strongly co‐adapted, that they function as a modular unit, and that this co‐adaptation plays a large role in climatic adaptation suggests avenues for targeted genetic rescue emphasizing the fitness of particular mitonuclear genotypes. Managers should consider the possibility that functionally distinct foreign mt haplotypes could positively influence mean fitness of a target population. These functional variants might exist among other populations of the same species, but they might also be found only in different species. Many populations have suffered extreme erosion of mt diversity due to anthropogenic pressures, with some species being reduced to a single mt haplotype (e.g., Van Der Valk et al., 2018; Wisely et al., 2015). A novel mt genome might be intentionally introgressed because it has suffered less mutational accumulation than the native one, because it is thought to be more adaptive under emerging environmental conditions, or simply to increase the number and diversity of mt haplotypes on which selection can act.

Genetic rescue typically involves related populations within the same species (where mitonuclear compatibility is unlikely to be a major concern), and has proven to be a remarkably effective conservation strategy (Frankham, 2015; Novak et al., 2021; Ralls et al., 2020). A meta‐analysis of genetic rescue to‐date found that, contrary to the expectation of worse outbreeding depression in F2 than F1 hybrids, fitness increases were statistically no different and trended towards being greater in F2s than F1s, consistent with an effect of maternally inherited genes (Frankham, 2015, 2016). Thus, *within* species, the introduction of a foreign mt haplotype might be associated with fitness benefits.

The risk of outbreeding could be evaluated by considering levels of genetic distance in the mt genome as a proxy for the degree of potential incompatibility, and population genetic studies already use putatively neutral markers to make management recommendations. Unlike differentiation in neutral markers, however, genetic incompatibilities are predicted to accrue at a speed equal to or greater than the square of the time since divergence (Matute et al., 2010; Orr, 1995). This might make neutral nuclear markers an exceptionally poor proxy for the consequences of introducing foreign mitochondria. Instead, managers could give particular weight to measures of functional differentiation in mt and N‐mt genes, including the ratio of nonsynonymous to synonymous substitutions and/or the ratio of radical to conservative amino acid changes. These approaches, as well as protein modeling, facilitate a priori predictions of mitonuclear incompatibility and are discussed in more detail in Appendix S2.

If functionally important mitonuclear variability exists, managers should select individuals with mt and N‐mt genes most likely to be compatible with those of the target population (Havird et al., 2016). While females are often introduced for their easier integration into social systems and greater impact on population growth rates, females bring foreign mitochondria with the potential for mitonuclear incompatibilities (Havird et al., 2016) or with potentially deleterious mt variants (Ochoa et al., 2017). The introduction of only males does not completely eliminate the possibility of mitonuclear incompatibility, as males will carry N‐mt alleles that could be incompatible with native mt haplotypes. However, by avoiding the introduction of a second mt haplotype, the number of potential inter‐population or interspecific incompatibilities is reduced by half. In admixed populations with mt and N‐mt alleles from both parental species, incompatibilities can occur with either mt haplotype (Moran et al., 2022).

While fears of outbreeding depression have often been overstated in the case against genetic rescue (Frankham, 2015, 2016; Frankham et al., 2011; Ralls et al., 2020), genetic incompatibilities can be expected when moving genes between different species. If genetic rescue is going to be attempted with the mt genome of a different species, then mitonuclear incompatibilities are a potentially significant form of outbreeding depression to be considered (Burton & Barreto, 2012; Hill, 2017; Ma et al., 2016; Moran et al., 2022). Such incompatibilities may manifest as reduced fitness in F1 and especially F2 hybrids (Burton et al., 2006; Ellison et al., 2008; Niehuis et al., 2008), which have a 25% chance of being homozygous for mitonuclear incompatibility at any involved locus. In the wild, hybridization would be followed by sorting amongst hybrid genotypes for those with compatible combinations due to natural selection. This entails a period of reduced mean population fitness due to genetic incompatibilities (transient outbreeding depression) as a necessary precursor to the expected increase in fitness due to rescue by the novel adaptive alleles (Aitken & Whitlock, 2013; Hamilton & Miller, 2016; Pereira et al., 2021). A population needs to be large enough to avoid extinction during this phase. Over subsequent generations in the hybrid swarm, the decay of linkage disequilibrium and backcrossing to the native population will create some hybrids with relatively little foreign ancestry besides the mt haplotype and key N‐mt genes. If the foreign mt haplotype has a net‐benefit to the population, it would spread with little further intervention.

### 4.2 Conservation Mitonuclear Replacement (CmNR) as an alternative to traditional genetic rescue

**FIGURE 1:**
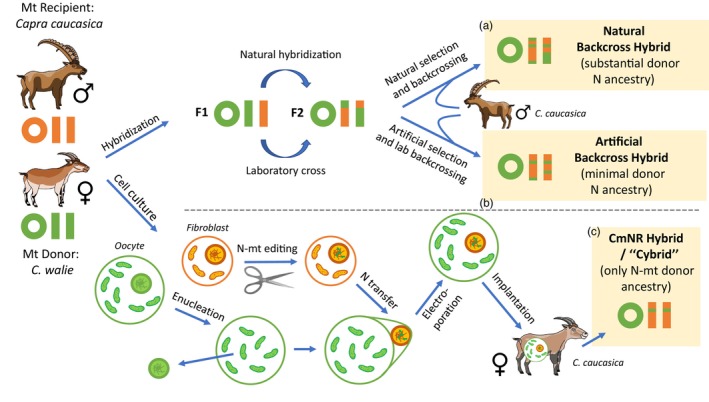
Three methods for introgressing mitochondrial genotypes while maintaining mitonuclear compatibility. The example species are the tropical Walian Ibex (*Capra walie*) as a mitochondrial donor and the endangered, temperate Western Tur (*C. caucasica*) as a recipient, which might benefit from a more warm‐adapted haplotype within its very limited range. To create a natural backcross hybrid (a), a female *C. walie* mt donor (green chromosomes; circular = mt, rectangular = nuclear) is introduced and naturally hybridizes with a wild male *C. caucasica*, creating F1 hybrids. These hybrids reproduce with themselves, creating F2 hybrids. Natural selection among F2 hybrids for mitonuclear compatibility, combined with backcrossing to parental‐species males, eventually creates hybrids with compatible N‐mt genes but with substantial tracts of donor nuclear (N) ancestry. To create an artificial backcross hybrid (b), initial F1 and subsequent F2 hybridization occurs in the lab, followed by artificial selection for hybrids not only with compatible mitonuclear genotypes but with minimal donor nuclear ancestry. To create a CmNR hybrid or “cybrid” (c), genetic editing and advanced reproductive technologies are used instead. A donor oocyte (large, green) is enucleated, removing its nucleus. A recipient somatic cell (small, orange) is edited at key N‐mt loci for mitonuclear compatibility. The somatic cell nucleus is then transferred into the membrane of the enucleated oocyte and fused by electroporation. The cybrid zygote is then implanted into a recipient species female for gestation. The resulting offspring have no donor species nuclear ancestry except at key compatibility loci that have been edited. Ibex drawings by Nat Jennings.

Traditionally, genetic rescue is attempted by releasing animals to interbreed in the wild, or, where outbreeding depression is a potential concern, crossing them first in a captive setting (e.g., Pavlova et al., 2023). While either of these approaches might be considered to introgress a divergent mitochondrial haplotype, they suffer from serious drawbacks—namely, a large investment in resources, the use of many individuals, a transitory reduction in mean fitness, and the introgression of off‐target nuclear ancestry that alters the recipient population's phenotype and evolutionary trajectory beyond the desired intervention. These hurdles may be avoided through the use of existing biotechnologies. Here, I describe a novel approach which I call Conservation Mitonuclear Replacement (CmNR; Figure 1), which combines nuclear transfer and genetic engineering to create a hybrid embryo with the nuclear genome of one species, the mitochondria of another, and the elimination of potential mitonuclear incompatibilities. Substantial technical detail, including many necessary caveats and refinements, is provided in Appendices S1 and S2. I then discuss the potential of natural and facilitated hybridization to achieve the same goals and their drawbacks relative to CmNR from a conservation perspective.

CmNR would combine reproductive biotechnologies and genetic engineering techniques to overcome mitonuclear incompatibilities and allow the replacement of a population's mt genome with that from a divergent population or a related species while eliminating many of the risks and unintended consequences of hybridization. Below, I discuss two potential approaches at different stages of technological feasibility. The first approach would rely on transferring mitochondria from a donor species to a recipient while editing the recipient's N‐mt loci. This approach should be achievable with current technologies, but the applicability, utility and accuracy of the technique for conservation will depend on continuing advances in assisted reproductive techniques (Appendix S1) and the prediction of mitonuclear incompatibilities (Appendix S2). The second approach would rely on directly editing mt loci, an emerging technology that may or may not prove fruitful. This technique would also be challenging because adaptive mt substitutions would need to be predicted a priori rather than relying on a donor mt haplotype that was naturally selected. Throughout, I refer to the species of conservation concern as the “recipient” and the species supplying novel mitochondria as the “donor,” despite the fact that in the case of the first technique, it is the nucleus of the recipient species which is being transferred into the cytoplasm of the “donor.”

The most realistic technique at present for introducing foreign mt haplotypes while maintaining mitonuclear compatibility would be to combine elements of interspecies somatic cell nuclear transfer (iSCNT) and mt replacement therapy (MRT) with targeted nuclear genetic editing of mitonuclear compatibility loci. Both iSCNT and MRT involve the transfer of nuclear chromosomes into enucleated cells to create a chimeric embryo. In conservation, iSCNT has long been pursued as a method to clone endangered or extinct species from frozen cell lines by transferring the somatic nucleus of one species into the cytoplasm of a closely‐related species' egg cell (Bolton et al., 2022; Gambini et al., 2022; Loi et al., 2011; Mastromonaco et al., 2014). While interspecific cloning has often been unsuccessful, a large degree of the difficulty derives from mitonuclear incompatibility between donor and recipient (Dobler et al., 2014; Loi et al., 2011; Mastromonaco et al., 2007; Wisely et al., 2015 and references therein). In clinical settings, MRT has recently seen advances and approvals as a technique to eliminate mt disease—the so‐called “three‐parent baby” approach, wherein haploid nuclear material is transferred into the cytoplasm of a healthy mt donor oocyte prior to fertilization (Adashi & Cohen, 2018; Hyslop et al., 2016; Kang et al., 2016; Kit et al., 2022; Reinhardt et al., 2013). For CmNR, CRISPR‐based gene editing of the nuclear material of the mt‐recipient species would first alter the sequences of important N‐mt genes to match those of the mt donor species. Then, using iSCNT or MRT methods (see Appendix S1), edited nuclear material would be transferred into the enucleated cytoplasm of the mt donor species' oocyte or zygote, which would reprogram the nucleus to begin embryonic development. Thus, in theoretically one generation, mt replacement could be achieved while maintaining mitonuclear compatibility, alleviating a source of negative iSCNT/MRT complications. This combination of existing technologies could reduce many of the potential negative consequences of natural or facilitated introgression by eliminating the transfer of unwanted nuclear material and minimizing the duration of captive breeding schemes. In particular CmNR, avoids disrupting the myriad non‐mt aspects of the phenotype that may be locally adapted, a major concern when facilitating adaptation through hybridization (Aitken & Whitlock, 2013).

There are two main technical challenges to such an approach, which are discussed in the supplements. The first pertains to assisted reproductive technologies (ARTs); while great advances have been made for model organisms and agriculturally important species, ARTs are still relatively limited for many of the taxa most likely to need CmNR. For instance, large, opaque oocytes and amniotic eggs make the usual procedures for nuclear transfer inapplicable to birds and reptiles (Appendix S1). Many ARTs will need refinement for the particular biology of the species in question. The second major challenge is the prediction and identification of important N‐mt alleles to target for genetic editing. While still in its infancy, the ability to anticipate mitonuclear incompatibilities is growing rapidly through experimental advances, structural modeling, and statistical genetics (Appendix S2). If progress on both fronts continues advancing, the major hurdles to implementation—as with other conservation biotechnologies—may prove to involve funding, regulation, and public reception.

The second approach to achieve CmNR would involve directly editing the mt genome, which would eliminate the need for mt replacement and expand the number of compatible assisted reproductive technologies. Mt base editing is currently relatively limited but is likely to advance substantially in the future (Silva‐Pinheiro & Minczuk, 2022; see Appendix S2). Synthesis of novel mtDNAs is also possible (Gibson et al., 2010), and their uptake by transformation followed by selective degradation of native haplotypes might provide a simpler route to engineered mitochondria (see Silva‐Pinheiro & Minczuk, 2022). However, de novo mt editing will be constrained by the difficulty of anticipating which mt genes to edit, because conservationists will have to predict adaptive variants a priori rather than rely on the consequences of millennia of natural selection (Appendix S2). The major area in which mt genetic editing or whole mitogenome synthesis is most likely to be useful is in re‐introducing extinct mt haplotypes that may carry adaptive variation that has been lost (see below).

### 4.3 Advantages of CmNR over traditional genetic rescue

CmNR can overcome several serious drawbacks of traditional genetic rescue techniques when it comes to intentionally introgressing a novel mt haplotype. Hybridization in the wild is uncertain, and entails the diversion of reproductive effort away from conspecifics in a recipient population that may already be small and/or endangered. If the populations are sufficiently divergent, there will be not only the expected mitonuclear incompatibilities but possibly hundreds of nuclear‐nuclear genetic incompatibilities that will need to be purged before mean fitness can increase (e.g., Schumer et al., 2014). A small population may not be able to survive this transitory reduction in mean fitness from novel genetic incompatibilities. Uninterrupted natural hybridization will also allow the introgression of many genes without important mitonuclear interactions, including the potential introduction of alleles that are poorly adapted for local conditions (Harris et al., 2019). A final difficulty with natural hybridization is that it requires foreign individuals to encounter and successfully mate with native individuals under natural conditions. Mitonuclear genotypes may be linked to sexual signals (Hill, 2011; Hill et al., 2019) and, because mt divergence between species can create intrinsic reproductive isolation, sexual signals are expected to diverge to prevent maladaptive hybridization (Hill, 2017). This might make it particularly unlikely that the recipient recognizes and chooses to mate with the donor species where meaningful mt differentiation is present.

Unnatural or laboratory conditions can induce species to hybridize that rarely or never hybridize in the wild and can ameliorate selection on hybrids, partially overcoming these difficulties. By genotyping hybrid offspring and backcrossing them to the recipient species, desired genes can be selectively introgressed while reducing the genetic input of the donor species. In the case of the American chestnut, this technique has been used to create hybrids with minimal Chinese chestnut (*C. mollissima*) ancestry (~6%) including the desired blight‐resistance genes. Here, an easy genotype screen was provided by the application of blight to seedlings and selective propagation of the survivors (Steiner et al., 2017; Worthen et al., 2010). However, screens for mitonuclear compatibility are less straightforward. The primary functional metric that can detect hybrid mitonuclear disfunction, high‐resolution respirometry, typically requires sacrificing the individual to obtain mitochondria‐rich tissues in sufficient quantities (e.g., Moran et al., 2022), though for larger animals a muscle biopsy will suffice. Other metabolic phenotypes, like metabolic rate, might indicate a measure of mt function, but with the confounding influence of many unrelated nuclear loci.

While facilitated hybridization in the lab might overcome some difficulties of natural hybridization in the wild, it involves a large investment in husbandry, with potentially hundreds or thousands of individuals kept over many generations. Even with highly‐accurate screens, the end result will still be hybrids with non‐negligible foreign ancestry that does not contribute to mitonuclear compatibility and may alter the phenotype in undesirable ways. In the case of hybrid American chestnuts, for instance, even the small amount of Chinese chestnut ancestry has changed the ecological properties of the trees and made them poorer competitors in North American forests than their forebearers (Newhouse & Powell, 2021). Due to the polygenic basis of natural pathogen resistance, further reductions in foreign ancestry are unlikely. By contrast, a transgenic American chestnut, which has only the addition of one foreign gene for blight tolerance, retains its full complement of adaptive native alleles (Newhouse & Powell, 2021). Biotechnologies like CmNR offer the potential to achieve the desired introgression in one generation, with no expensive captive breeding scheme, no reduction in mean population fitness, and no undesirable nuclear ancestry. Thus, provided that progress continues in assisted reproductive technologies (Appendix S1) and in predicting mitonuclear interactions (Appendix S2), it may sometimes be more desirable to use CmNR to introduce desired mitonuclear genetic variation than natural or facilitated hybridization.

BOX 1 **Mitonuclear introgression on São Tomé**

An illustrative case of mitonuclear co‐introgression occurred on the island of São Tomé in the Gulf of Guinea, which might reflect demographic and/or climatic factors. The widespread lowland African fly *Drosophila yakuba* inhabits savannas and grasslands up to around 1450 m, while above this zone, from 1150 to 1900 m, the endemic rainforest species *D. santomea* is found. In the middle elevations where they overlap, a hybrid zone exists (Llopart et al., 2005a), and introgression of the mt genome and limited regions of the nuclear genome from *D. yakuba* into *D. santomea* has occurred (Llopart et al., 2005b). *D. yakuba* and *D. santomea* are sister species, and *D. santomea* probably descended from an initial island colonization by *D. yakuba* 400,000 years ago, with *D. yakuba* returning to the island during Portuguese colonization. Through hybridization and back‐crossing, the mt genome of *D. yakuba* has come to fully replace that of *D. santomea* in the upper mountains (Llopart et al., 2014). In tandem with this introgression, three nuclear subunits of cytochrome c oxidase (COX/Complex IV) that complex intimately with mt proteins have also introgressed upslope (Beck et al., 2015). As suggested by Llopart et al. (2014), this may be a case where an endemic mt genome with high mutational load due to small effective population size (a problem likely to plague many mountaintop or island species; Box 2) was replaced by a genome from a much larger population with a lower mutational load. However, it may also be the case that this novel mitonuclear combination was favored over the native *D. santomea* genotype because of increasing average temperatures over the last 150 years, or the widespread deforestation that followed colonization (Siebinga, 2018). In any case, it appears that the lowland *D. yakuba* mitogenome is currently more fit at all elevations, and it is worth considering whether its introgression into *D. santomea*, facilitated by co‐introgression of N‐mt genes, has enabled *D. santomea* to persist on the mountaintop rather than being outcompeted by *D. yakuba*. It will be interesting to observe whether nuclear gene flow increases over time now that a potential mitonuclear incompatibility has been neutralized, and whether the relative positions of the two species shift with further climate change.

## 5 APPLICATIONS OF CmNR

**FIGURE 2:**
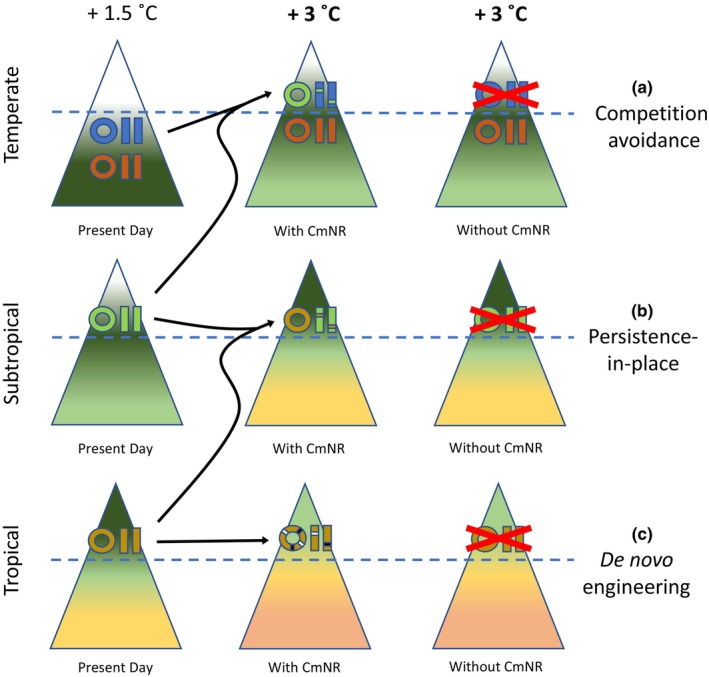
Three models applying Conservation Mitonuclear Replacement (CmNR) on isolated mountaintops. Species of differing colors are represented by mitochondrial genomes (rings) and nuclear genomes (paired rectangular chromosomes). Mountains at three latitudes have three elevational niches, each of differing temperatures at differing elevations: two below and one above some critical oxygen (O_2_) threshold (dashed blue line). Mountains in the left column are in the present day, at roughly 1.5°C of committed warming above historical baseline temperatures, while mountains in the middle and right columns are in the future after 3°C of warming and depict outcomes with and without CmNR, respectively. (a) Competition avoidance: the blue temperate‐zone species is prevented from migrating upward with increasing temperatures by oxygen limitation above the O_2_ threshold. CmNR introduces a mitogenome and N‐mt gene edits from a related species in the subtropics which lives higher and is adapted to the same temperatures, allowing the blue species to track rising temperatures. In the absence of CmNR, the blue species goes extinct because it fails to migrate past the oxygen barrier and is out‐competed by the orange species moving up from below. (b) Persistence‐in‐place: a subtropical species is prevented from migrating upwards to track temperatures because of a lack of higher‐elevation habitat on the mountain. CmNR from a related tropical species occurring at the same elevation allows persistence in the face of warming. In the absence of CmNR the species goes extinct due to unsuitable temperatures. (c) De novo engineering: a tropical species likewise is prevented from tracking temperatures due to a lack of high‐elevation habitat. No related species occurs at more‐tropical latitudes or warmer elevations. In the future, introduction of mt & N‐mt edits de novo might allow creation of a novel mitonuclear genotype better suited to warmer conditions. In the absence of CmNR the species goes extinct due to unsuitable temperatures.

There are many ecological scenarios in which CmNR might prove useful. I highlight them in Table 2, and in Figure 2 I depict several specific scenarios related the challenges global warming poses for species living at the top of elevation gradients with nowhere to migrate to (Freeman et al., 2018; Marris, 2007; Moritz et al., 2008; Urban, 2018). This scenario, discussed further in Box 2, represents, one of the most tractable cases for conceptualizing, piloting, and executing CmNR. Similar scenarios can be envisioned with regard to latitude rather than elevation, with polar species occupying a “mountaintop” with nowhere left to migrate to. Other species for which migration is limited and CmNR may be useful include those with inherent dispersal limitations or for which natural features (deserts, water barriers, etc.), unchanging environmental gradients, or anthropogenic land use change make movement impossible. Island species, for instance, have almost no capacity for migration, and possess inherently low effective population sizes that might cause dangerous mutational loads in mtDNA (Woolfit & Bromham, 2005). Some species also have cultural values that would be lost if they could not survive in situ, such as alpine ungulates important to local hunting traditions (Figure 2, Table 2). Managers will need to decide, based on ecological and cultural values, whether facilitated migration or facilitated adaptation makes more sense in these scenarios.

**TABLE 2:** Examples of potential Conservation Mitonuclear Replacement scenarios and taxa which might benefit from them.

Scenario	Recipient species/pop	Recipient range	Potential threat	Donor species/pop	Donor range	Notes
*Persistence‐in‐place*						
Replace high elevation mt with lower latitude, comparable elevation mt	Western tur (*Capra caucasica*)	Caucasus Mountains, 800–4000 m	Limited potential for upward migration	Walian ibex (*C. walie*)	Ethiopian highlands, 2300–4000 m	Figure 1. Assisted reproductive technologies already developed for iSCNT in *Capra* (Alabart et al., 2009)
Replace high‐elevation mt with lower elevation, comparable latitude mt	Sira Tanager (*Tangara phillipsi*)	Isolated Cerros del Sira, Peru, 1300–2200 m	No potential for upward migration	Dotted tanager (*T. varia*)	Cordillera Azul, Peru, 400–1100 m	Maximum elevation of Cerros del Sira is 2250 m
	Quito whorl‐tail iguana (*Stenocercus guentheri*)	Ecuadorian Andes, 2230–3700 m	Unsuitably warm temps and fragmentation‐limited dispersal	Huancabamba whorl‐tail iguana (*S. huancabambae*) or La Granja whorl‐tail iguana (*S. arndti*)	N. Peruvian Andes, 200–1500 m (*S. huancabambae*) or 2000–2320 m (*S. arndti*)	*S. guentheri* predicted to undergo local extinction by 2080 due to thermal traits (Andrango et al., 2016)
Replace high‐latitude mt with extinct low‐latitude mt	Boreal Woodland Caribou (*Rangifer tarandus caribou*)	Boreal forest and Northern Rockies	Unsuitably warm temps at S range margin	Selkirk Mountains population (Southernmost, extirpated); extinct E US populations	Northern Idaho; Minnesota, Wisconsin, Michigan, Maine	One Selkirk individual survives in captivity (Moskowitz, 2019)
	Grizzly Bear (*Ursus arctos horribilis*)	Alaska through Northern Rockies	Unsuitably warm temps at S range margin	California Grizzly (*U. a. californicus*); Mexican Grizzly (*U. a. horribilis/nelsoni*)	California; Central Mexico	Lack of hibernation in extinct populations could indicate metabolic adaptation to milder winters
Replace lowland tropical mt with more arid‐adapted mt	Brazil Nut (*Bertholettia excelsa*); Brazil nut pollinators (*Eulaema* spp., *Xylocopa* spp., etc.)	Amazonia	Aridification/savannization	Other Lecythidaceae; other Euglossine bees	Across South America	Threatened by drought‐fire feedback and land conversion; orchids used by bees are potential third candidates in the symbiosis for CmNR
Replace low‐acidity mt with high‐acidity mt	Reef‐building corals (*Acropora* spp., *Porites* spp., etc.)	Shallow tropical waters worldwide	Ocean acidification limiting ability to form calcium skeletons	High‐pCO2 adapted congeners; ancient DNA with adaptive significance	Naturally occurring CO2 seeps; similar locations under past conditions	Elevated mt function helps seep‐living corals sclerify at high pCO2 (Agostini et al., 2021). Likely mt effect on thermotolerance as well (Dixon et al., 2015). Recovery of 6000 y old aDNA: (Scott et al., 2022)
Engineer de‐novo warm‐adapted mt haplotypes	American Pika (*Ochotona princeps*), southern populations	Southern Rockies	Unsuitable temps and limited capacity for upward migration	No more warm‐adapted species or populations exist		Figure 2c. Candidate for assisted migration (Chen, Grossfurther, et al., 2022; Chen, Jiang, et al., 2022)
	Tuatara (*Sphenodon puctatus*)	Predator‐free islands of New Zealand	Unsuitable temps, skews in sex ratio of offspring	No related species of any kind (250 mya split from squamates)		Candidate for assisted migration (Miller et al., 2012)
*Adaptation‐to‐migrate*						
Replace low‐elevation mt with high	Royal cinclodes (*Cinclodes aricomae*)	Isolated *Polylepis* woodlands, Central Andes, 3700–4600 m	Possible environmental limitation of upward habitat tracking	White‐bellied cinclodes (*C. paliatus*)	High‐elevation bogs, W Central Peru, 4400–5500 m	*Polylepis* likely to contract and move upslope with climate change (Caballero‐Villalobos et al., 2021)
	Pale‐billed woodpecker (*Campephilus guatemalensis*)	Lowland Central America & Mexico	Possible environmental limitation of upward habitat tracking	Extinct Imperial Woodpecker (*C. imperialis*)	Mexican & Guatemalan highlands	Figure 2a. *C. imperialis* mitogenome sequenced (Anmarkrud & Lifjeld, 2017)
*Restoration of lost diversity*						
Recover lost mt haplotypes	Black‐footed ferret (*Mustela nigripes*)	Isolated populations in former range	Only one mt haplotype in living individuals	Black‐footed ferret (historical samples); Domestic ferret (*M. putorius furo*)	Entire Great Plains; Domesticated in Europe	Figure 2a. CmNR could resurrect extinct mt haplotypes or overcome incompatibilities with domestic surrogates (Wisely et al., 2015)
	Crested ibis (*Nipponia nippon*)	Isolated populations in former range	Extremely low genetic diversity in living individuals	Crested ibis (historical samples)	Across East Asia	Ancient DNA recovered from many museum specimens (Feng et al., 2019)
De‐extinction	Asian elephant (*Elephas maximus*)	South & Southeast Asia	Potential incompatibility in engineered Asian elephant oocytes with mammoth N‐mt genes; warmer temperatures than during the Pleistocene	Extinct woolly mammoth (*Mammuthus primigenius*)	Holarctic	Mitonuclear incompatibilities between *Loxodonta* spp. (Roca, 2019); possible mammoth mt adaptation (Ngatia et al., 2019)

MtDNA haplotypes themselves might play a large role in determining whether species can migrate, such as facilitating upward tracking of temperature by a montane species into a zone where it faces hypoxia (Figure 2a). In general, a major consideration in applying CmNR (and assisted evolution in general) is whether it is desirable to attempt to enable a species to persist in place (Figure 2b) or to track favorable conditions (and potentially avoid competition) by migrating (Figure 2a). The former will probably only be an option for species lacking parapatric congeneric competitors that replace them on the warm edge of their range, or decisions may need to be made in different locales about which congener to preserve. One area in which enabling persistence‐in‐place may be useful is in the vast lowland tropics, where species face grim prospects for dispersal and biodiversity will likely suffer “lowland attrition” (Colwell et al., 2008). The issue is compounded by the fact that tropical lowland species typically live at or near their upper thermal limits (Deutsch et al., 2008; Huey et al., 2009). While there exist no warmer, equally wet habitats from which to draw mt donor species, much of the tropics is aridifying (Franchito et al., 2012), and mt haplotypes from tropical dryland species might prove useful to persistence‐in‐place. In the Amazon, for instance, Brazil nut trees (*Bertholletia excelsa*) are an umbrella species for conservation and an important non‐timber forest product threatened by agricultural land conversion and a deforestation‐drying feedback loop that could shift the region from forest to savanna (Coe et al., 2013; Franchito et al., 2012; Jansen et al., 2021). Land conversion around trees also impairs pollination, while climate change threatens to shrink the range of the tree's unique pollinators (Sales et al., 2021). CmNR could be used either to improve the tolerance of the trees themselves or their pollinators to ongoing land use and climate changes, thus safeguarding the essential economic and biodiversity benefits of Brazil nut agroforestry (Table 2). Other keystone species for which persisting in place is important include reef‐building corals, and mt variation might hold the key to facilitating adaptation to heat waves and ocean acidification (Agostini et al., 2021; Dixon et al., 2015; Table 2).

If no suitable mt haplotypes exist, a key source of adaptive mt haplotypes might prove to be extinct species and populations. For instance, a number of northern temperate species such as brown bears (*Ursus arctos*) and caribou (*Rangifer tarandus*) have seen the extinction of populations on their southern range margin, removing potentially warm‐adapted mt haplotypes that could be crucial for adapting to climate change (Table 2). In other cases, highland species such as the imperial woodpecker (*Campephilus imperialis*) have gone extinct, taking with them haplotypes that could have allowed congeners to successfully migrate upslope into the mountains (Table 2). The restoration of extinct mt haplotypes could be useful not only for directly facilitating adaptation, but for restoring mt genetic diversity to populations that have suffered population bottlenecks. Extinct haplotypes could replace extant ones with high mutational load, or simply provide more potentially‐adaptive variation on which selection can act. Recovery of ancient mtDNA sequences is possible from frozen remains, specimens, or continuous deposits (Anmarkrud & Lifjeld, 2017; Rogaev et al., 2006; Scott et al., 2022) and can lead to synthetic mtDNA plasmids that may be able to be injected for uptake by recipient mt populations (Gibson et al., 2010; Silva‐Pinheiro & Minczuk, 2022). Native recipient mtDNAs could then be targeted for degradation (Jackson et al., 2020; Silva‐Pinheiro & Minczuk, 2022; see Appendix S1).

Aspects of CmNR can also be applied during traditional cloning of nuclear material for conservation or rewilding purposes, alleviating complications caused by mitonuclear incompatibilities. For instance, endangered species cloning often relies on congeneric surrogates because the reproductive resources of the target species are unavailable or limited. However, the efficacy of interspecific cloning is low, and incompatibilities between the target nuclear material and the surrogate's mitochondria are a likely culprit (Mastromonaco et al., 2007; Wisely et al., 2015; Appendix S1). If cloned species are destined to be produced with foreign mitochondria, nuclear editing of key incompatibility loci could not only increase the efficacy of cloning but improve the lifetime fitness of clones. This would also allow the use of surrogates further removed from the target species, an advantage for evolutionarily distinct species with no living congeners. Such a technique could be used in de‐extinction and rewilding efforts like that of the woolly mammoth (*Mammuthus primigenius*), which may rely on surrogates of a different genus (Asian elephant, *Elephas maximus*). Current initiatives aim to selectively edit elephant nuclear DNA to make it more mammoth‐like (DeFrancesca, 2021), but mammoth mtDNA may be key to metabolic adaptation to the arctic (Ngatia et al., 2019). The resurrection of mammoth mitochondria by transformation or mt editing could go hand‐in‐hand with the editing of key N‐mt loci in elephants to recreate the mammoth metabolic phenotype Table 2.

The general principles of CmNR – environmental co‐adaptation of organellar and nuclear genomes, replacement and/or co‐editing of genes in both genomes for facilitated adaptation, and the importance of these co‐adapted gene sets in reproductive isolation – apply to plants as well as animals. Mitonuclear coevolution is important in plants (Hanson & Bentolila, 2004; Havird et al., 2017) and, like mitochondria, the chloroplast has a reduced set of genes that complex intimately and co‐evolve with nuclear genes (Ceriotti et al., 2022; Forsythe et al., 2021), influence adaptation to heat, drought, and other stressors (Song et al., 2021; Yoo et al., 2019), and contribute to reproductive isolation (Postel & Touzet, 2020; Sambatti et al., 2008). However, distinct aspects of plant biology make it hard to generalize some of the conclusions of this paper (see Sloan, 2015), including their differing methods of mt transmission, the expanded gene content of plant mt genomes, the interactions between multiple symbiotic organelles, the methods of plant reproductive biotechnology, the mechanisms of plant environmental adaptation and distribution, the nature of competition between related plants, and the propensity of plants to hybridize. Specific proposals for Conservation Mitonuclear Replacement in plants—or, Conservation Cytonuclear Replacement, when including chloroplasts—will benefit from future botanical attention. Furthermore, the principals of CmNR apply beyond endosymbiotic organelles, to any intracellular or intimately‐interacting symbiont that might be manipulated for a conservation purpose. For instance, applications to modify intracellular insect symbionts like *Wolbachia* to control invasive species, or efforts to replace coral symbionts like *Symbiodinium* for thermal tolerance, will benefit from the consideration and potential editing of key compatibility loci in the host (see Hoadley et al., 2019; Wall et al., 2020).

BOX 2 **The escalator to extinction and CmNR on warming mountains**

Mountains contain a disproportionate share of Earth's biodiversity (Myers et al., 2000) and their dynamic topography has been the primary driver of diversification in some groups of animals (Igea & Tanentzap, 2021). However, many species are moving upslope to track their preferred temperatures, and those near the tops of mountains may have nowhere else to go (Chen et al., 2011; Feeley et al., 2011; Forero‐Medina et al., 2011; Freeman & Class Freeman, 2014; Freeman et al., 2018). Even if a species' physiology might allow it to persist, it may be outcompeted by congeners moving upslope from below (Urban et al., 2012). The mountaintop extinctions predicted to follow—the “escalator to extinction”—have already been documented locally and are a major threat to global biodiversity (de la Fuente et al., 2022; Freeman et al., 2018; Hoffmann et al., 2020; La Sorte & Jetz, 2010; Urban, 2018). In fact, in some regions, up to 58% of species could be driven extinct by upslope range shifts (Thomas et al., 2004), and elevational limitation can explain up to 97% of the variation in climate‐driven extinction risk in some groups (Sekercioglu et al., 2008). Species at any point on the elevational gradient may also be subjected to decreasing population sizes as they move upward due to the inverse relationship between land area and elevation (Iverson et al., 2024). Tropical species appear to be more closely tracking climate upslope than their temperate counterparts (Freeman et al., 2021); however, the rate at which species track climate typically lags behind the actual increase in temperatures (Chen et al., 2011; Forero‐Medina et al., 2011; Freeman et al., 2018). For tropical organisms in particular, evolutionary conservatism in their climatic niche will limit their ability to adapt to changing conditions (Linck et al., 2021). Because tropical species tend to have more narrow thermal tolerances (Janzen, 1967; Khaliq et al., 2014; McCain, 2009), their capacity for latitudinal dispersal is also limited, as the nearest cooler latitudes are far out of reach. If an environmental factor like hypoxia provides a hard limit on vertical dispersal (e.g., Spence et al., 2022), even tall mountains will provide little refuge and extinctions will be common (La Sorte & Jetz, 2010).

It has been suggested that the only hope for species facing extinction under these conditions is translocation to somewhere outside of the native range where conditions will be more suitable (Thomas, 2011); however, facilitated adaptation through CmNR might play a role in conserving endangered montane species in situ. First, species might be assisted in moving upwards by altering their tolerance to hypoxia, preventing them from being squeezed into extinction by species encroaching on their lower range margin (Figure 1a). The source of this augmented adaptive variation should ideally come from populations of the same genus at higher altitudes in more equatorial parts of the range, for reasons discussed in the main text. Several high‐elevation species have received adaptive introgression of hypoxia alleles (such as EPAS1 variants) from close relatives that were already living at elevation (Huerta‐Sánchez et al., 2014; Montejo‐Kovacevich et al., 2022; Wang et al., 2020; Wang, Ju, et al., 2021; Wang, Ore, et al., 2021), providing direct evidence that congeneric introgression can facilitate adaptation to altitude. Second, mountaintop species might be given greater tolerance for warm conditions, either allowing them to persist at native elevation beyond their thermal limits, or to remain competitive with warm‐adapted species moving up from below (Figure 1b). Remaining in situ could be important for species at the top of the gradient, or if a key resource is expected to persist in place. If tolerance to hypoxia is important, then the source of this adaptive mitonuclear variation would ideally come from populations at similar elevations but more equatorial latitudes, rather than from those lower on the same mountain. This would also prevent the collapse of reproductive isolation from parapatric low‐elevation congeners (see section 6). Decisions about where to source novel mt haplotypes require intense eco‐physiological consideration and could have profound consequences for the effectiveness of CmNR.

## 6 ECOLOGICAL, EVOLUTIONARY, AND ETHICAL IMPLICATIONS OF CmNR

Because CmNR combines both facilitated adaptation and genetic engineering, CmNR projects require serious ecological, evolutionary, and ethical consideration, as well as meaningful public & stakeholder engagement to avoid negative consequences both for biodiversity and for perceptions of value. Any genetic intervention in wild populations can cause significant public anxiety or backlash, presents a moral hazard (by remediating the symptoms rather than the causes of biodiversity loss), and fundamentally alters our relationship to the natural world and the value commitments of conservation (Kohl et al., 2019; Redford et al., 2019; Sandler, 2020). Such tactics would seem to support a switch from *altering the environment* to suit organisms to *altering organisms* to suit their environment (though it is not necessary to abandon the first approach.) All relevant considerations about the practicality, safety, and desirability of manipulating genetic variation in wild organisms apply to CmNR, including off‐target genomic effects, hybridization with non‐target populations, unintended competitive interactions, unnatural population expansion, or unforeseen population fitness reduction and even extinction. Test cases need to be chosen carefully and evaluated thoroughly.

Recent debate has centered on the possibility of accidentally introducing deleterious variation during genetic rescue (see Ralls et al., 2018, 2020); however, in hundreds of genetic rescues to date, the consequences have been overwhelmingly positive, with little evidence of outbreeding depression (Chan et al., 2019; Frankham, 2015, 2016; Hirashiki et al., 2021). Mitonuclear thinking clarifies some of the important features that differentiate species, adapt them to their environments, and create reproductive isolation between them, helping to target gene flow, avoid outbreeding depression, and minimize alteration to gene pools. Additionally, there is a pervasive concern in conservation for the integrity of species, subspecies, and evolutionary units; traditional genetic rescue often aims to minimize genetic differences between donor and recipient populations, preserving their unique genetic and phenotypic attributes while still alleviating the deleterious effects of inbreeding (Harris et al., 2019; Ralls et al., 2018). Likewise, facilitated adaptation by hybridization or selective breeding is tailored towards minimizing genetic change in the target population (e.g., Steiner et al., 2017; Worthen et al., 2010). Although the public often perceives genetic engineering as “invasive” or “unnatural,” genetic engineering for conservation entails far less substantial modifications to species' genotypes and phenotypes than does hybridization or intraspecific genetic rescue (Newhouse & Powell, 2021). Thus, while decisions about hybridization may contain somewhat subjective choices about what makes a species or population worthy of distinction (Hirashiki et al., 2021), genetic engineering sidesteps these debates by changing only the necessary loci and leaving almost the entire genome and phenotype unaltered.

Despite the minimal genomic alteration entailed by techniques like CmNR, there may still be important downstream consequences that go beyond the intended fitness benefits. Co‐adapted mt and N‐mt alleles are hypothesized to differentiate species not simply as a consequence of evolution in isolation, but to represent the barrier loci actually creating intrinsic post‐zygotic isolation between them (Hill, 2017, 2019b). Multiple lines of evidence demonstrate the utility of a species concept based on (or incorporating) mitonuclear compatibility, including the importance of known interspecific mitonuclear incompatibilities (Jhuang et al., 2017; Ma et al., 2016; Moran et al., 2022; Trier et al., 2014; van der Heijden et al., 2019) and the concordance between mt barcodes and taxonomic species designations (Hill, 2020; Stoeckle & Thaler, 2014). Evidence also comes from the creation of ‘cybrid’ embryos (cytoplasmic hybrids), which are hybrids with a diploid nucleus from one source inserted into foreign cytoplasm (with foreign mitochondria); almost invariably, interspecific cybrids have reduced fitness relative to intraspecific ones (Bowles et al., 2007; Kenyon & Moraes, 1997; Loi et al., 2011; references in Ma et al., 2016; Appendix S1). It has been argued that mitonuclear discordance, either natural or anthropogenic, does not disrupt the identity of a species and the integrity of its lineage for the purposes of conservation (Novak, 2018). However, barrier loci—those that determine reproductive isolation between populations—have an outsized role to play in evolutionary processes (Elmer, 2019; Ravinet et al., 2017). While the impact on the exterior phenotype from changing a mitonuclear locus may be small, the loss of reproductive isolation from the removal of a barrier locus might have profound evolutionary consequences down the line.

If mitonuclear incompatibilities are a major cause of reproductive isolation, and mitonuclear interactions play an integral role in metabolic environmental adaptation, then mitonuclear phenotypes are so‐called “magic traits” (Gavrilets, 2004; Servedio et al., 2011) that cause reproductive isolation via divergent ecological adaptation. Parapatric species typically replace each other with a change in habitat or a semi‐permeable environmental barrier, and such traits might play a role in explaining how they maintain their respective genetic identities. Parapatry relies not on sufficient ecological differentiation for sympatric coexistence, nor on complete pre‐zygotic isolation, which parapatric species often lack, but on reciprocally higher fitness in their respective ranges and a partial barrier to gene flow. On an environmental cline, the border between two species' ranges theoretically occurs at the point where their fitness is equal. Often there is a narrow zone of sympatry, and hybrid zones, evidenced by gene flow that may be phenotypically cryptic, are extremely common in nature and occur at least transiently for many para‐ or sympatric species (Dagilis et al., 2021; Ottenburghs et al., 2017; Singhal et al., 2021; Taylor & Larson, 2019). It may be more the rule than the exception that neighboring species exchange some genes, with at least 25% of plants, 10% of animal species, and 20% of North American bird species known to hybridize (Grant & Grant, 1992; Mallet, 2005; Ottenburghs, 2019, 2021; Ottenburghs et al., 2015). The increasing incidence of hybridization as species' ranges shift (Chunco, 2014; Grabenstein & Taylor, 2018; Ottenburghs, 2021) reinforces the idea that reproductive isolation between parapatric species is only partial and that environmentally‐mediated competition sets their boundaries (see Martin & Ghalambor, 2023). If mitonuclear loci are some of the most important incompatibilities between parapatric species, then CmNR will allow for extensive gene flow between them; this could cause genetic swamping, particularly by the species on the warmer edge, and might simply collapse the two into one undifferentiated species only weakly adapted to conditions at either end of its range (Seehausen et al., 2008). For this reason, CmNR should be attempted with a donor that is allopatric with regards to the recipient, so accidental nuclear gene flow is an unlikely consequence. Mitonuclear incompatibility with a third species may be shared between two species due to hybridization and introgression (Moran et al., 2022; see also Sun et al., 2018), meaning that CmNR will not necessarily compromise isolation between the recipient species and neighboring parapatric species that are not the mt donor.

Allowing one species to persist while its ecosystem migrates ahead of it—or lags behind it—will be disruptive to the composition of natural communities and established ecological relationships. A major area for research into CmNR should involve the impacts of mt haplotypes on modulating competition between congeners and on ecological traits such as foraging rate. However, objections about “ecological integrity” misattribute the actual values of biodiversity and complexity that a technique like CmNR seeks to preserve (Rohwer & Marris, 2021). Not all members of a community will respond to climate change identically, and differential migration rates are already expected to cause widespread disruptions to community composition and function (de la Fuente et al., 2022; Kinlock et al., 2018; Lurgi et al., 2012; Moritz et al., 2008). CmNR is not a technique which could feasibly be applied to a whole community to freeze it in place; indeed, even if it could, this would hinder the migration of more warm‐adapted communities, leading to massive “boxcar effects” as migrating species pile up (Urban et al., 2012). Instead, the technique might conserve a particular population of a particular species in a particular situation while the remainder of its populations go extinct. Considerations of ecological function, evolutionary distinctiveness, and cultural significance should thus play a role in identifying CmNR candidates.

CmNR should be pursued in situations in which traditional conservation techniques have been explored and are not sufficient to prevent extinction. However, CmNR should not be seen only as a technique of last resort; rather, given the certainty of further climate change and the current state of species distribution modeling, we should be proactively identifying species that may need intervention and planning for the intended consequences of implementing it. Even under a severe emissions‐reduction scenario, a certain amount of warming is “locked in” by already‐emitted greenhouse gasses (IPCC, 2021; Zhou et al., 2021). Facing this inevitability, identification of future CmNR candidates will help target conventional conservation efforts to prevent species from needing such biotechnological interventions. A substantial number of species will still be threatened by rising temperatures and/or poor mt diversity that no traditional intervention can alleviate, whether from a lack of suitable translocation sites or a dearth of conspecific adaptive variation. Failure to plan for the possibility of CmNR would leave little time to intervene once populations reach a critical situation. Decision‐makers must weigh the certain costs of inaction against the usual concerns about “unintended consequences” in conservation interventions (Brister et al., 2021; Phelan, Baumgartner, et al., 2021; Phelan, Kareiva, et al., 2021). Under ongoing levels of greenhouse gas emissions, the worst imaginable unintended consequence of CmNR will likely pale in comparison to anticipated disruptions to natural communities.

## 7 PILOTING AND IMPLEMENTING CmNR

There are two important lines of inquiry that should be pursued to test the feasibility of CmNR. The first involves piloting the proposed combination of genetic editing and mitochondrial replacement to create healthy individuals; the second involves testing whether such individuals do indeed survive or migrate better in the face of environmental change and potential competition. Rodents are a good group in which to pilot biotechnological approaches due to the sophistication of mouse, rat, and vole models. As mentioned earlier, in *Myodes* voles, a potential case of adaptive mt replacement characterizes the expansion of the southern *M. glareolus* into harsher northern climes inhabited by *M. rutilus* (Boratyński et al., 2011). Future work could use whole‐genome sequencing across the hybrid zone to establish whether any nuclear loci co‐introgressed with the cold‐adapted *M. rutilus* mitotype. If so, a good proof of concept would be to attempt CmNR in the opposite direction, using warm‐adapted *M. glareolus* as the mt donor and *M. rutilus* as the recipient. Any loci identified as co‐introgressors during the natural hybridization could be edited to match the *M. glareolus* sequence, and the success of embryos with mt replacement against both edited and unedited nuclear backgrounds could be established. Finally, measuring the metabolic rate and lower critical temperatures of both native *M. rutilus* and those with introduced *M. glareolus* mitochondria could establish whether the intervention had the impact of increasing heat tolerance.

While insects might be unlikely candidates for CmNR, species such as *Drosophila* could provide a good model system for testing the ecological dimensions of mitonuclear combinations (see Box 1). Genetic manipulation is well‐developed in *Drosophila* and flies lend themselves to population‐level laboratory experiments. *Drosophila* species from different latitudes possess species‐specific differences in mt function that help explain both their upper and lower thermal tolerance limits (Jørgensen et al., 2023). Experiments could be conducted to establish whether the degree of mitonuclear matching among introgressed lines is relevant to thermal performance. Then, mesocosm experiments could test the ecological significance of mt replacements and the degree of mitonuclear matching. For instance, in a population raised with access to a thermal gradient, experiments could test whether mt replacement with or without editing of N‐mt loci affected the ability of flies to occupy parts of the gradient that were previously too hot. If two species were raised along a thermal gradient, CmNR could be performed on the species at the cool end of the gradient to improve thermal performance; then, by raising the temperature of the entire gradient, it could be seen whether CmNR allowed the cool‐adapted species to persist and resist competition by the warm‐adapted species.

The ideal trial candidate for CmNR in the wild would occur in several populations (for instance, isolated mountain ranges; see Box 2). CmNR can thus be tested in one population before impacting the entire species, and comparisons can be made between fitness in recipient and control populations. It is important to understand the nature of future conditions and the specific threat; if increasing temperature is combined with decreasing precipitation, for instance, then a mt donor population must be well adapted to both sets of conditions. Ideally, a trial could be carried out with CmNR populations in locations both with and without warm‐edge congeneric competitors. Feasibility studies for CmNR should also identify whether key resources needed by a species will continue to exist in the range that CmNR might enable it to occupy.

Subtropical mountains may represent the most important arena for CmNR, because temperate species can more easily move across latitudes, while tropical species have few congeners that could be more warm‐adapted. Surveys should be undertaken to understand the distribution of native genetic variation across the species' range and to ascertain whether a simpler solution might be the facilitated migration of conspecifics (e.g., Chen, Grossfurther, et al., 2022; Chen, Jiang, et al., 2022). If hybrid individuals already exist between a potential donor and recipient species, then these should be screened for mitonuclear genotypes, and their translocation to the site might be a simpler way to effect mitonuclear co‐introgression. In species with multiple disjunct populations, these approaches could be compared to CmNR to assess effectiveness as a pilot study.

When planning for application in the wild, there will be innumerable ecological studies that could be conducted to try to predict how CmNR might influence competitive, trophic, and other interactions with the surrounding community of species that will not migrate, or the new species that could migrate into the range of the CmNR‐recipient species. I recommend beginning CmNR trials while such ecological studies are still underway, given that conservation already suffers from a severe knowledge‐action gap (Arlettaz et al., 2010; Cook et al., 2013). Conservation interventions need to scale‐up dramatically and rapidly to meet contemporary challenges, tipping the balance from the overcautious inaction of the Precautionary Principal to the “intended consequences” of beneficial intervention (Brister et al., 2021; Phelan, Baumgartner, et al., 2021; Phelan, Kareiva, et al., 2021); adaptive management, or “learning‐while‐doing,” is the only policy strategy commensurate with this change (Brister et al., 2021; Funk et al., 2019; Keith et al., 2011; Runge, 2011).

To implement CmNR, characterization of mitonuclear diversity and modeling of potential incompatibilities should first occur, followed by creation of edited cell lines and cybrid embryos. Finally, a captive‐rearing facility near the native range can begin to raise cybrid individuals and prepare them for release into the wild. Funds should be secured ahead of time for raising and releasing cybrid individuals and for long‐term monitoring to determine whether they persist and increase in numbers. If successful, the technique should then be considered for a short‐list of suitable species facing near‐term peril. Because many CmNR candidates are facing extinction in the wild, captive populations might be established long before testing and approval of CmNR. Such “arc” populations could provide a source of genetic material for CmNR, and the re‐establishment of wild populations could be attempted in the future with individuals better suited to new conditions. This situation is in fact ideal for CmNR, since there are no individuals left in the wild to interbreed with CmNR individuals and dilute the donor mt haplotype.

## 8 CONCLUSION

Many populations are likely to suffer from mt haplotypes which lead to poor fitness, whether due to bottlenecks that remove potentially adaptive haplotypes and allow deleterious variants to accumulate, or due to anthropogenic changes such as global warming. Mitonuclear genotypes play a significant role in environmental tolerance and represent an integrative metabolic suite that has been naturally transferred between species in a way that facilitates adaptation. I propose a novel form of assisted evolution which I call Conservation Mitonuclear Replacement, combining approaches from mt replacement therapy and interspecies somatic cell nuclear transfer with conventional nuclear genetic engineering. This technique would allow replacement of a species' mt genome with that of a related species that is either intrinsically more fit or that is better adapted to changing environmental conditions. Future advances might also enable synthetic or even directly edited mt genomes to be introduced, although the latter presents greater theoretical difficulties in elucidating adaptive substitutions a priori.

When considering the certainty and magnitude of continuing anthropogenic global change, managers must consider the expected harms of inaction alongside the potential harms of action (Brister et al., 2021; Phelan, Baumgartner, et al., 2021; Phelan, Kareiva, et al., 2021). Climate change threatens millions of species and might remove up to 10% of global biodiversity under a scenario of business‐as‐usual conservation (Thomas et al., 2004; Urban, 2015). A significant degree of warming cannot be avoided even by immediate emissions reductions, and existing conservation techniques will likely be inadequate to preserve many important species. Faced with this threat, it seems inevitable that multiple avenues of facilitated adaptation will be needed. CmNR represents a proposal for evolutionary rescue at a limited number of loci with known adaptive significance, following a natural pattern of adaptive introgression of the mt genome that appears to have already facilitated the expansion and persistence of many species.

## CONFLICT OF INTEREST STATEMENT

The author declares no conflict of interest.

## Supporting information


Appendix S1.

Appendix S2.

## Data Availability

Data sharing is not applicable to this article as no datasets were generated or analyzed during the current study.
