# Supplementary material for: Conservation Mitonuclear Replacement: Facilitated mitochondrial adaptation for a changing world
Source: Evol Appl. 2024 Mar 10;17(3):e13642. doi: 10.1111/eva.13642 (PMC10925831; doi:10.1111/eva.13642)
Supplement: Supplementary file 1 — Appendix S1. [file EVA-17-e13642-s002.pdf]

Supplement 1: Nuclear editing and mitochondrial transfer in Conservation Mitonuclear Replacement

Before nuclear transfer, it will be necessary to identify N-mt compatibility loci (see Supplement 2) and edit them with standard nuclear genetic editing techniques such as CRISPR. In practice, it will probably be necessary to propagate cell lines after gene-editing to ensure the success of the procedure and lack of off-target effects before moving on to nuclear transfer. However, such interspecies cellular hybrids suffer a unique obstacle never encountered in natural hybridizations: they are homozygous for every potential mitonuclear incompatibility at once. For instance, these 'cybrid' embryos—meaning the hybrid formed by a diploid nucleus inserted into a foreign mt background—between highly differentiated mouse subspecies can fail to develop due to the severity of mitonuclear incompatibilities (Ma et al., 2016). Despite the presence of mitonuclear incompatibilities in post-editing cells, however, even issues that eventually cause embryonic lethality may not always compromise the ability of cybrid cells to grow and divide up to a point (Ma et al., 2016; Moran et al., 2022). Intergeneric cybrids, such as those between humans and gorillas, can create proliferating cell lines with detectable respiration (Barrientos, Kenyon, & Moraes, 1998; Kenyon & Moraes, 1997). If it appears that substantial mitonuclear incompatibility is causing issues in cell lines between genetic editing and nuclear transfer, one potential solution would be to edit only a portion of N-mt genes before mt replacement. If half of the nuclear genes were edited before nuclear transfer, and half after in a second round, then the total number of incompatibilities at any point in time would be halved.

If nuclear editing has been successful, mt replacement would proceed by transferring a recipient nucleus into the enucleated cytoplasm of a mt-donor cell (Sato et al., 2005; Taylor & Turnbull, 2005). In the clinical context this is typically performed from one haploid oocyte to

another before fertilization; however, the goal here is to transfer the diploid nucleus of a somatic cell or zygote to an enucleated oocyte or zygote (e.g., Sato et al., 2005). Nuclear editing either before or after nuclear transfer to an oocyte is a standard method of genetic engineering in livestock (Galli & Lazzari, 2021; Sheets et al., 2016). One advantage of performing editing first in CmNR is that the edits are achieved in a cell with functioning mitochondria and that nuclear transfer restores any function compromised by the edits. If nuclear transfer were performed first, editing would occur in a cell with compromised mitonuclear compatibility, which might cause complications. Performing editing in somatic cells rather than in the valuable, limited number of cybrid zygotes also simplifies the process of creating cell lines to check for editing efficiency. If resources permit, however, the creation of large numbers of zygotes is one of the principal determinants of success in SCNT endeavors (Wisely, Ryder, Santymire, Engelhardt, & Novak, 2015).

SCNT has been successful in mammals, fishes, amphibians, and insects, but genetic manipulation is lagging in birds and non-avian reptiles because access to the nucleus is difficult in large oocytes and zygotes (Rasys et al., 2019; Seok et al., 2008). One workaround developed for birds involves transferring primordial germ cells (PGCs) from one species into the developing embryo of another, creating chimeras with foreign gonadal tissue (Seok et al., 2008). CRIPSR/Cas-9 nuclear editing in isolated PGCs has been achieved (Dimitrov et al., 2016), and with mt replacement at this stage, male and female chimeras of the recipient species could be created which produce offspring of their own species with the mitochondria of another. In reptiles, editing and SCNT might be accomplished via surgical access to immature oocytes (Rasys et al., 2019). Assisted reproductive techniques like these will need continued refinement before CmNR is feasible in some taxa.

One potential complication of SCNT techniques is that they are not necessarily designed to reduce *heteroplasmy*—the co-mingling of two mt haplotypes in one cell or body. In fact, increased mt carryover with the nucleus may be favorable to SCNT outcomes (Jiang et al., 2011; Loi, Modlinski, & Ptak, 2011; Ma et al., 2016; Yan et al., 2010), demonstrating the importance of mitonuclear compatibility for fitness. In a heteroplasmic population of mtDNAs, certain mtDNAs may be favored and replicate faster due to greater compatibility with the nuclear replication machinery, even if they causes disease in the organismal phenotype (Kang et al., 2016; Yamada, Akashi, Ooka, Miyado, & Akutsu, 2020). This can cause even low initial levels of heteroplasmy to increase throughout development (Hyslop et al., 2016; Kang et al., 2016; Sato et al., 2005). The spindle transfer technique deployed during metaphase II of meiosis in MRT can reduce heteroplasmy to below 1% (Kang et al., 2016), and a similar technique could be applied in the metaphase of mitosis to capture the diploid spindle-chromosome complex. Pronuclear transfer after fertilization offers another technique for low-carryover (<2%) mt replacement in diploid cells (Hyslop et al., 2016). At these low levels, the minor mitotype is often purged and becomes undetectable both in the somatic and germline tissue (Yamada et al., 2020). Prior nuclear editing should also remove any potential mitonuclear incompatibilities that could favor recipient species mtDNA.

In general, the state of assisted reproductive technology is likely to be the main technological constraint on any CmNR project. Refinements will be necessary before the approach outlined here can become generally applicable, including perhaps the further reduction of heteroplasmic mt carryover. The techniques above are typically inconsistent at delivering the lowest levels of heteroplasmy possible (Yamada et al., 2020), so it may be necessary to test embryos for donor mtDNA content before proceeding with implantation. If mt carryover remains

an issue, techniques have been developed to selectively degrade recipient mtDNA, effectively eliminating heteroplasmy after mt replacement (Fan et al., 2022; Reddy et al., 2015). If prior enucleation of the donor oocyte removed a substantial amount of mitochondria, then supplementation of cytoplasm from another donor cell may facilitate successful blastulation (Bowles, Campbell, & St. John, 2007). It is also important that the donor and recipient cells are synchronized in their cell cycles so that nuclear reprogramming and embryonic development may occur, and that mitonuclear compatibility at this stage is sufficient to allow nuclear activation of mt transcription and replication (Bowles et al., 2007; Wisely et al., 2015).

Works Cited

Barrientos, A., Kenyon, L., & Moraes, C. T. (1998). Human xenomitochondrial cybrids: Cellular models of mitochondrial complex I deficiency. *Journal of Biological Chemistry*, *273*(23), 14210–14217. https://doi.org/10.1074/jbc.273.23.14210

Bowles, E. J., Campbell, K. H. S., & St. John, J. C. (2007). Nuclear transfer: Preservation of a nuclear genome at the expense of its associated mtDNA genome(s). *Current Topics in Developmental Biology*, *77*(06), 251–290. https://doi.org/10.1016/S0070-2153(06)77010-7

Dimitrov, L., Pedersen, D., Ching, K. H., Yi, H., Collarini, E. J., Izquierdo, S., … Leighton, P. A. (2016). Germline gene editing in chickens by efficient crispr-mediated homologous recombination in primordial germ cells. *PLoS ONE*, *11*(4), 1–10. https://doi.org/10.1371/journal.pone.0154303

Fan, X. Y., Guo, L., Chen, L. N., Yin, S., Wen, J., Li, S., … Luo, S. M. (2022). Reduction of mtDNA heteroplasmy in mitochondrial replacement therapy by inducing forced mitophagy. *Nature Biomedical Engineering*, *6*(4), 339–350. https://doi.org/10.1038/s41551-022-00881-7

Galli, C., & Lazzari, G. (2021). Current applications of SCNT in advanced breeding and genome editing in livestock. *Reproduction*, *162*, F23–F32. https://doi.org/10.1093/oso/9780198862086.003.0020

Hyslop, L. A., Blakeley, P., Craven, L., Richardson, J., Fogarty, N. M. E., Fragouli, E., … Herbert, M. (2016). Towards clinical application of pronuclear transfer to prevent mitochondrial DNA disease. *Nature*, *534*(7607), 383–386. https://doi.org/10.1038/nature18303

Jiang, Y., Kelly, R., Peters, A., Fulka, H., Dickinson, A., Mitchell, D. A., & St. John, J. C. (2011). Interspecies somatic cell nuclear transfer is dependent on compatible mitochondrial

DNA and reprogramming factors. *PLoS ONE*, *6*(4). https://doi.org/10.1371/journal.pone.0014805

Kang, E., Wu, J., Gutierrez, N. M., Koski, A., Tippner-hedges, R., Agaronyan, K., … Don, P. (2016). Mitochondrial replacement in human oocytes carrying pathogenic mitochondrial DNA mutations. *Nature*, *540*(218). https://doi.org/10.1038/nature20592

Kenyon, L., & Moraes, C. T. (1997). Expanding the functional human mitochondrial DNA database by the establishment of primate xenomitochondrial cybrids. *Proceedings of the National Academy of Sciences*, *94*, 9131–9135.

Loi, P., Modlinski, J. A., & Ptak, G. (2011). Interspecies somatic cell nuclear transfer: A salvage tool seeking first aid. *Theriogenology*, *76*(2), 217–228. https://doi.org/10.1016/j.theriogenology.2011.01.016

Ma, H., Marti Gutierrez, N., Morey, R., Van Dyken, C., Kang, E., Hayama, T., … Mitalipov, S. (2016). Incompatibility between nuclear and mitochondrial genomes contributes to an interspecies reproductive barrier. *Cell Metabolism*, *24*(2), 283–294. https://doi.org/10.1016/j.cmet.2016.06.012

Moran, B. M., Payne, C. Y., Powell, D. L., Iverson, E. N. K., Banerjee, S. M., Langdon, Q. K., … Schumer, M. (2022). A lethal genetic incompatibility between naturally hybridizing species in mitochondrial complex I. *BioRxiv*, *v2*, 1–45. https://doi.org/10.1101/2021.07.13.452279

Rasys, A. M., Park, S., Ball, R. E., Alcala, A. J., Lauderdale, J. D., & Menke, D. B. (2019). CRISPR-Cas9 gene editing in lizards through microinjection of unfertilized oocytes. *Cell Reports*, *28*(9), 2288-2292.e3. https://doi.org/10.1016/j.celrep.2019.07.089

Reddy, P., Ocampo, A., Suzuki, K., Luo, J., Bacman, S. R., Williams, S. L., … Izpisua Belmonte, J. C. (2015). Selective elimination of mitochondrial mutations in the germline by genome editing. *Cell*, *161*(3), 459–469. https://doi.org/10.1016/j.cell.2015.03.051

Sato, A., Kono, T., Nakada, K., Ishikawa, K., Inoue, S. I., Yonekawa, H., & Hayashi, J. I. (2005). Gene therapy for progeny of mito-mice carrying pathogenic mtDNA by nuclear transplantation. *Proceedings of the National Academy of Sciences of the United States of America*, *102*(46), 16765–16770. https://doi.org/10.1073/pnas.0506197102

Seok, J. K., Jin, W. C., Sun, Y. K., Kyung, J. P., Tae, M. K., Young, M. L., … Jae, Y. H. (2008). Reproduction of wild birds via interspecies germ cell transplantation. *Biology of Reproduction*, *79*(5), 931–937. https://doi.org/10.1095/biolreprod.108.069989

Sheets, T. P., Park, C.-H., Park, K.-E., Powell, A., Donovan, D. A., & Telegu, B. P. (2016). Somatic cell nuclear transfer followed by CRIPSR-Cas9 microinjection results in highly efficient genome editing in cloned pigs. *International Journal of Molecular Sciences*, *17*(2031). https://doi.org/10.3390/ijms17122031

Taylor, R. W., & Turnbull, D. M. (2005). Mitochondrial DNA mutations in human disease. *Nature Reviews Genetics*, *6*(5), 389–402. https://doi.org/10.1038/nrg1606

Wisely, S. M., Ryder, O. A., Santymire, R. M., Engelhardt, J. F., & Novak, B. J. (2015). A road map for 21st century genetic restoration: Gene pool enrichment of the black-footed ferret.

Journal of Heredity, *106*(5), 581–592. https://doi.org/10.1093/jhered/esv041

Yamada, M., Akashi, K., Ooka, R., Miyado, K., & Akutsu, H. (2020). Mitochondrial genetic drift after nuclear transfer in oocytes. *International Journal of Molecular Sciences*, *21*(16), 1–13. https://doi.org/10.3390/ijms21165880

Yan, Z. H., Zhou, Y. Y., Fu, J., Jiao, F., Zhao, L. W., Guan, P. F., … Zeng, F. (2010). Donor-host mitochondrial compatibility improves efficiency of bovine somatic cell nuclear transfer. *BMC Developmental Biology*, *10*. https://doi.org/10.1186/1471-213X-10-31
