# Supplementary material for: Conservation Mitonuclear Replacement: Facilitated mitochondrial adaptation for a changing world
Source: Evol Appl. 2024 Mar 10;17(3):e13642. doi: 10.1111/eva.13642 (PMC10925831; doi:10.1111/eva.13642)
Supplement: Supplementary file 2 — Appendix S2. [file EVA-17-e13642-s001.pdf]

## Supplement 2: *A priori* detection of mitonuclear incompatibilities

Conservation mitonuclear replacement could theoretically be performed in a manner agnostic as to the identities of key mitonuclear compatibility loci; by editing all N-mt genes to match the sequence of the mt donor, all potential mitonuclear incompatibilities would be accounted for. Despite the vast reduction in the difficulty of gene-editing that CRISPR/Cas systems have enabled, however, the prospect of editing potentially thousands of genes is daunting. It is also doubtful whether we know the true number of mt-interacting nuclear loci, and omnigenic models propose that essentially all genes have minor interactions with all traits (Boyle, Li, & Pritchard, 2017). Luckily, the available evidence, much of it detailed in the main text, suggests that the number of mitonuclear incompatibilities with strong impacts on hybrid fitness is manageable. New advances in CRISPR allow editing of 25-30 loci simultaneously (Campa, Weisbach, Santinha, Incarnato, & Platt, 2019; Yuan & Gao, 2022), and multiple rounds of editing can be performed in the unlikely event that the number of important loci exceeds this. In the most well-characterized mitonuclear incompatibility network, just two nuclear genes (encoding Complex I subunits of the ETS) demonstrate strong incompatibilities with one mt haplotype, while 2-3 others could have weak interactions; there is also an incompatibility involving the other mt haplotype and an unknown number and identity of nuclear genes (Moran et al., 2022).

The key impediment to realistic CmNR is identifying *a priori* which nuclear loci need to be edited. How can the fitness effects of novel mitonuclear incompatibilities be predicted and assessed without access to adult hybrids of every particular genetic combination? There are several existing techniques that can be employed, and with the accumulation of data on mitonuclear incompatibilities in natural hybrids and laboratory crosses our power to predict potential incompatibilities is growing. Approaches to identifying mitonuclear incompatibility

loci are the same as those for identifying any Bateson-Dobzhansky-Muller incompatibility (BDMI) causing reproductive isolation, of which they are simply a subset (Burton & Barreto, 2012), with the added expedient that the number of potentially interacting alleles is reduced. A general pipeline will look something like this: first, using a phylogeny of the potential donor, the recipient, and related taxa, all derived nucleotide substitutions in the mt genome and the core subset of ~1100 N-mt genes are identified, with the subset that cause nonsynonymous amino-acid substitutions identified for special consideration. If population genomic data is available, nuclear loci that are outliers of differentiation by $F_{ST}$ and other metrics can also be scanned for mt-associated functions. Candidate genes can be identified as the subset of directly-interacting genes with a derived substitution in both partners or two derived substitutions in one gene (regardless of whether the two substitutions were derived in the same or different species; see Cattani & Presgraves, 2009). Among this group, there could be nuclear substitutions at loci that interact little with mt genes, but are important for nuclear-nuclear compatibility among N-mt genes (e.g., the OXPHOS supernumerary proteins). From these subsets of candidate genes, structural modeling can be used to identify which substitutions are in a position to actually interact with one another, and whether the substitutions they possess are expected to significantly alter the structure and function of interacting components in ways that could be detrimental to fitness. This general approach, examined in more detail below, could reduce a list of thousands of substitutions to only a handful which are strong candidates to cause mitonuclear incompatibility during mt replacement. Some specific candidate genes are identified in Table 1.

Theory predicts that genetic incompatibilities will arise first in the fastest evolving loci (Maheshwari & Barbash, 2011; Presgraves, 2010), that they may be under positive selection (Brideau et al., 2006), and may occur at loci with branch-length asymmetries between species

(Dagilis, Kirkpatrick, & Bolnick, 2019). By examining metrics such as the $d_N/d_S$ ratio, which measures excess nonsynonymous substitutions, the degree of potentially consequential differentiation in proteins can be assessed. The ratio of radical-to-conservative amino acid substitutions ($D_r/D_c$) may also be useful. Evolutionary rate covariation between mt and nuclear genes has proven powerful in detecting intimately-interacting genes, and demonstrates that OXPHOS proteins with sites that physically interact have the tightest rate covariation and thus the highest potential for incompatibilities (Havird, Maclaine, Tressel, & Weaver, 2021; Piccinini et al., 2021; Yan, Ye, & Werren, 2019). These sequence variants can then be projected onto structural models to determine how specific amino acid substitutions are likely to impact conformation and stability between interacting molecules (Hill et al., 2019; Jhuang, Lee, & Leu, 2017; Scott et al., 2011; Sunnucks, Morales, Lamb, Pavlova, & Greening, 2017). To give two theoretical examples from OXPHOS, a substitution that increases the distance between two components of the electron transport system only slightly could impact the ease of quantum tunneling and jeopardize the flow of electrons; a substitution that creates novel bonds could change conformational states and jeopardize the pumping of protons (Rak et al., 2016). This kind of modeling can also identify changes in the affinity of transcriptional machinery for mtDNA or of aminoacyl tRNA-synthetases for specific mt-tRNAs. While it would be difficult to predict the overall fitness effects from such substitutions, it may be possible to distinguish the top candidates for severe incompatibility and focus efforts on these. It may also be possible to perform a degree of phenotyping on post-editing cells. For instance, if editing has variable penetrance and not all targeted genes are edited in every cell, cells will vary in the degree of mitonuclear compatibility they express. High-throughput systems such as the Seahorse XF plate-based respirometer (Agilent, Santa Clara, CA) might be able to detect important differences in

single-cell respiration, with the most compromised cybrid cells representing those with the highest proportion of important mitonuclear compatibility loci successfully edited.

Overall, while the predictive detection of any genetic incompatibilities is in its infancy, the field is progressing rapidly, while the number of empirically documented incompatibilities for reference is increasing (Table 1). Mitonuclear incompatibilities in particular have several characteristics that make their detection and characterization simple relative to nuclear-nuclear incompatibilities. For one, the reduced size and limited gene content of the animal mitogenome vastly limits the number of potential incompatibilities. The number of nuclear genes thought to directly interact with mt genes is also relatively limited (around 200, see Table 1 for some), while a larger but still manageable number (around 1100) are imported into the mitochondria and have at least indirect interactions with mt genes (Burton & Barreto, 2012). In addition, all mitonuclear incompatibilities are by definition tri-allelic, involving one mt allele and two nuclear alleles, and have six potential states (aAA, aAB, aBB, bAA, bAB, bBB), while nuclear-nuclear incompatibilities involve four alleles and nine potential states (aaAA, aaAB, aaBB, abAA, abAB, abBB, bbAA, bbAB, bbBB). This reduces the statistical power needed to detect mitonuclear incompatibilities from population genomic data. In animals, mt genes usually evolve faster than nuclear genes, up to 20 times faster in vertebrates (Allio, Donega, Galtier, & Nabholz, 2017). This fact may increase the likelihood that isolated populations will evolve mitonuclear incompatibilities (Maheshwari & Barbash, 2011; Presgraves, 2010), especially when considering the branch-length asymmetry that may be present between mt and nuclear genes and the increased likelihood of negative epistasis when uniparental loci are involved (Dagilis et al., 2019). Despite rapid evolution, the tertiary structures of core mt proteins are highly conserved, facilitating inference across taxa (da Fonseca, Johnson, O’Brien, Ramos, & Antunes, 2008).

These factors, which might contribute to the commonality of mitonuclear incompatibilities as BDMIs (Burton & Barreto, 2012), increase the likelihood that ameliorating only one or several incompatibilities via nuclear editing could allow successful CmNR. It is worth noting that as human mt replacement therapy gains acceptance, the screening approach discussed here can also prevent potential disease-causing mitonuclear incompatibilities, which segregate at low levels in human populations (Gershoni et al., 2014; Levin, Blumberg, Barshad, & Mishmar, 2014).

There is one developing innovation that could drastically change the prospects for genetic engineering of mitonuclear compatibility: mt genetic editing. With the ability to edit mt sequences directly, one might edit the native mitogenome of a species to better adapt it to the changing climate rather than replace its mitogenome with that of a congener. Direct mt DNA editing, alongside nuclear editing, would negate the need for nuclear transfer and the negative complications that can induce. Mt genetic editing could also be used to revive extinct haplotypes that might have adaptive benefits, drawing sequences from museum specimens or ancient tissues and editing them into existing mt haplotypes.  However, mt genetic editing is currently in its infancy compared to nuclear editing. Existing tools are limited to making only particular kinds of base edits (Mok et al., 2020; Silva-Pinheiro et al., 2022). In addition to the difficulties of targeting and importing editing machinery through mt membranes, edits must be successfully performed in nearly all of the hundreds or thousands of mitochondria in the cell. Edits that slightly reduce replication efficiency may be lost from the population, due to selection at the level of competing mt haplotypes. However, edits that improve mitonuclear compatibility might also quickly spread to fixation.

The main difficulty in mt genetic editing is that one must predict the absolute fitness effects of novel mutations in the environment, rather than simply trying to predict whether

particular genetic interactions are deleterious. In using an existing mt haplotype, natural selection operating at multiple levels (Rand, 2001) has derived both mt and nuclear sequences that are well-adapted to the environment and to each other. One need only import the foreign mitochondria and determine which nuclear genes have co-evolved tightly enough to require editing. If editing the mitochondria, however, one needs to know *a priori* either which mt substitutions from a related species are responsible for adaptation, or what entirely novel substitutions could be induced to create adaptation. The same must be determined for nuclear substitutions, while maintaining compatibility between the two genomes. While our genomic and structural analyses are increasingly able to predict good or poor fit between interacting gene products, it seems doubtful that we are near to predicting the lifetime fitness effects of novel mutations on the whole organism. The technique could, however, be used in lieu of mt replacement to introduce the substitutions that separate a recipient from a donor. This would allow, for instance, the introduction of extinct mt haplotypes into living populations, either simply to increase mt diversity or for specific environmental adaptations they are believed to harbor. If mt editing remains difficult, synthesis of plasmids with extinct haplotypes followed by transformation and selective degradation of native mt haplotypes might provide another route to reviving ancient haplotypes with adaptive benefits (Gibson, Smith, Hutchison, Venter, & Merryman, 2010; Jackson, Turnbull, Minczuk, & Gammage, 2020; Silva-Pinheiro & Minczuk, 2022).

## Works Cited

Allio, R., Donega, S., Galtier, N., & Nabholz, B. (2017). Large variation in the ratio of mitochondrial to nuclear mutation rate across animals: Implications for genetic diversity and the use of mitochondrial DNA as a molecular marker. *Molecular Biology and*

*Evolution*, *34*(11), 2762–2772. https://doi.org/10.1093/molbev/msx197

Boyle, E. A., Li, Y. I., & Pritchard, J. K. (2017). An expanded view of complex traits: from polygenic to omnigenic. *Cell*, *169*(7), 1177–1186. https://doi.org/10.1016/j.cell.2017.05.038

Brideau, N. J., Flores, H. A., Wang, J., Maheshwari, S., Wang, X., & Barbash, D. A. (2006). Two Dobzhansky-Muller genes interact to cause hybrid lethality in *Drosophila*. *Science*, *314*(5803), 1292–1295. https://doi.org/10.1126/science.1133953

Burton, R. S., & Barreto, F. S. (2012). A disproportionate role for mtDNA in Dobzhansky-Muller incompatibilities? *Molecular Ecology*. https://doi.org/10.1111/mec.12006

Campa, C. C., Weisbach, N. R., Santinha, A. J., Incarnato, D., & Platt, R. J. (2019). Multiplexed genome engineering by Cas12a and CRISPR arrays encoded on single transcripts. *Nature Methods*, *16*(9), 887–893. https://doi.org/10.1038/s41592-019-0508-6

Cattani, M. V, & Presgraves, D. C. (2009). Genetics and lineage-specific evolution of a lethal hybrid incompatibility between *Drosophila mauritiana* and its sibling species. *Genetics*, *181*, 1545–1555. Retrieved from https://doi.org/10.1534/genetics.108.098392

da Fonseca, R. R., Johnson, W. E., O'Brien, S. J., Ramos, M. J., & Antunes, A. (2008). The adaptive evolution of the mammalian mitochondrial genome. *BMC Genomics*, *9*, 1–22. https://doi.org/10.1186/1471-2164-9-119

Dagilis, A. J., Kirkpatrick, M., & Bolnick, D. I. (2019). The evolution of hybrid fitness during speciation. *PLoS Genetics*, *15*(5), 1–21. https://doi.org/10.1371/journal.pgen.1008125

Gershoni, M., Levin, L., Ovadia, O., Toiw, Y., Shani, N., Dadon, S., … Mishmar, D. (2014). Disrupting mitochondrial-nuclear coevolution affects OXPHOS complex I integrity and impacts human health. *Genome Biology and Evolution*, *6*(10), 2665–2680. https://doi.org/10.1093/gbe/evu208

Gibson, D. G., Smith, H. O., Hutchison, C. A., Venter, J. C., & Merryman, C. (2010). Chemical synthesis of the mouse mitochondrial genome. *Nature Methods*, *7*(11), 901–905. https://doi.org/10.1038/nmeth.1515

Havird, J. C., Maclaine, K. D., Tressel, L., & Weaver, R. J. (2021). Causes and consequences of mitochondrial mutation rate variation. *Society for Molecular Biology and Evolution Virtual Meeting*, (July 3-8), Poster. Retrieved from https://sites.cns.utexas.edu/sites/default/files/havird/files/havird_smbe_poster_portrait_052621.pdf?m=1622209758

Hill, G. E., Havird, J. C., Sloan, D. B., Burton, R. S., Greening, C., & Dowling, D. K. (2019). Assessing the fitness consequences of mitonuclear interactions in natural populations. *Biological Reviews*, *94*(3), 1089–1104. https://doi.org/10.1111/brv.12493

Jackson, C. B., Turnbull, D. M., Minczuk, M., & Gammage, P. A. (2020). Therapeutic manipulation of mtDNA heteroplasmy: A shifting perspective. *Trends in Molecular Medicine*, *26*(7), 698–709. https://doi.org/10.1016/j.molmed.2020.02.006

Jhuang, H., Lee, H., & Leu, J. (2017). Mitochondrial–nuclear co-evolution leads to hybrid incompatibility through pentatricopeptide repeat proteins. *EMBO Reports*, *18*(1), 87–101.

https://doi.org/10.15252/embr.201643311

Levin, L., Blumberg, A., Barshad, G., & Mishmar, D. (2014). Mito-nuclear co-evolution: The positive and negative sides of functional ancient mutations. *Frontiers in Genetics*, *5*(DEC), 1–11. https://doi.org/10.3389/fgene.2014.00448

Maheshwari, S., & Barbash, D. A. (2011). The genetics of hybrid incompatibilities. *Annual Review of Genetics*, *45*, 331–355. https://doi.org/10.1146/annurev-genet-110410-132514

Mok, B. Y., de Moraes, M. H., Zeng, J., Bosch, D. E., Kotrys, A. V, Raguram, A., … Liu, D. R. (2020). A bacterial cytidine deaminase toxin enables CRISPR-free mitochondrial base editing. *Nature*. https://doi.org/10.1038/s41586-020-2477-4

Moran, B. M., Payne, C. Y., Powell, D. L., Iverson, E. N. K., Banerjee, S. M., Langdon, Q. K., … Schumer, M. (2022). A lethal genetic incompatibility between naturally hybridizing species in mitochondrial complex I. *BioRxiv*, *v2*, 1–45. https://doi.org/10.1101/2021.07.13.452279

Piccinini, G., Iannello, M., Puccio, G., Plazzi, F., Havird, J. C., & Ghiselli, F. (2021). Mitonuclear coevolution, but not nuclear compensation, drives evolution of OXPHOS complexes in bivalves. *Molecular Biology and Evolution*, *38*(6), 2597–2614. https://doi.org/10.1093/molbev/msab054

Presgraves, D. C. (2010). The molecular evolutionary basis of species formation. *Nature Reviews Genetics*, *11*(3), 175–180. https://doi.org/10.1038/nrg2718

Rak, M., Bénit, P., Chrétien, D., Bouchereau, J., Schiff, M., El-Khoury, R., … Rustin, P. (2016). Mitochondrial cytochrome c oxidase deficiency. *Clinical Science*, *130*(6), 393–407. Retrieved from https://doi/org/10.1042/CS20150707

Rand, D. M. (2001). The units of selection on mitochondrial DNA. *Annual Review of Ecology, Evolution, and Systematics*, *32*, 415–448. Retrieved from http://dx.doi.org/10.1016/B978-0-323-03354-1.50092-4

Scott, G. R., Schulte, P. M., Egginton, S., Scott, A. L. M., Richards, J. G., & Milsom, W. K. (2011). Molecular evolution of cytochrome c oxidase underlies high-altitude adaptation in the bar-headed goose. *Molecular Biology and Evolution*, *28*(1), 351–363. https://doi.org/10.1093/molbev/msq205

Silva-Pinheiro, P., & Minczuk, M. (2022). The potential of mitochondrial genome editing. *Nature Reviews Genetics*, *23*(April). https://doi.org/10.1038/s41576-021-00432-x

Silva-Pinheiro, P., Nash, P. A., Van Haute, L., Mutti, C. D., Turner, K., & Minczuk, M. (2022). In vivo mitochondrial base editing via adeno-associated viral delivery to mouse post-mitotic tissue. *Nature Communications*, *13*(1), 1–9. https://doi.org/10.1038/s41467-022-28358-w

Sunnucks, P., Morales, H. E., Lamb, A. M., Pavlova, A., & Greening, C. (2017). Integrative approaches for studying mitochondrial and nuclear genome co-evolution in oxidative phosphorylation. *Frontiers in Genetics*, *8*(MAR), 1–12. https://doi.org/10.3389/fgene.2017.00025

Yan, Z., Ye, G., & Werren, J. H. (2019). Evolutionary rate correlation between mitochondrial-

encoded and mitochondria-associated nuclear-encoded proteins in insects. *Molecular Biology and Evolution*, *36*(5), 1022–1036. https://doi.org/10.1093/molbev/msz036

Yuan, Q., & Gao, X. (2022). Multiplex base- and prime-editing with drive-and-process CRISPR arrays. *Nature Communications*, *13*(1), 1–13. https://doi.org/10.1038/s41467-022-30514-1
